# Comparing human pediatric immune responses to primary infection with dengue, chikungunya and Zika viruses

**DOI:** 10.3389/fimmu.2025.1679566

**Published:** 2025-11-19

**Authors:** Lewis E Tomalin, Sandra Bos, Rafael Fenutria, Yitong Chen, Seunghee Kim-Schulze, Adeeb H Rahman, Angel Balmaseda, Ana Fernandez-Sesma, Eva Harris, Mayte Suaréz-Fariñas

**Affiliations:** 1 Department of Population Health and Science Policy, Icahn School of Medicine at Mount Sinai, New York, NY, United States; 2 Division of Infectious Diseases and Vaccinology, School of Public Health, University of California, Berkeley, Berkeley, CA, United States; 3 Department of Microbiology, Icahn School of Medicine at Mount Sinai, New York, NY, United States; 4 Department of Genetics and Genomic Sciences, Icahn School of Medicine at Mount Sinai, New York, NY, United States; 5 Human Immune Monitoring Center, Icahn School of Medicine at Mount Sinai, New York, NY, United States; 6 Laboratorio Nacional de Virología, Centro Nacional de Diagnóstico y Referencia, Ministerio de Salud, Managua, Nicaragua; 7 Sustainable Sciences Institute, Managua, Nicaragua

**Keywords:** arbovirus, immune response, mass cytometry (CyTOF), cytokine, multiomic analyses, flavivirus, alphavirus

## Abstract

**Background:**

The four dengue virus serotypes (DENV1-4), Zika virus (ZIKV) and chikungunya virus (CHIKV) have similar epidemiology and transmission cycles and are the most prevalent arthropod-borne viruses in humans, with half the world’s population at risk of infection. Although these infections share overlapping clinical presentations, the immune mechanisms that distinguish these infections, particularly in children, remain poorly defined. We aimed to characterize the immune responses to DENV, ZIKV, and CHIKV in a pediatric population and to define the specific immune signatures associated with each virus.

**Methods:**

We characterized the immune responses to DENV, ZIKV and CHIKV by measuring cytokine/chemokine/growth-factor profiles in plasma/serum samples, and immune-cell profiles in peripheral blood mononuclear cells, collected from children during acute (~1–3 and ~4-6 days) primary infection with DENV1/DENV3 (n=32), ZIKV (n=50) or CHIKV (n=45), and during infection recovery (~14–25 days).

**Results:**

The innate immune responses to CHIKV and DENV were similar in terms of acute cytokine concentrations and monocyte frequencies. The innate immune response to ZIKV was mild, and the adaptive response was delayed, showing much lower concentrations of inflammatory cytokines and delayed T cell/B cell activation. Overall, the immune response to CHIKV and DENV were most similar than DENV and ZIKV, despite DENV and ZIKV belonging to the same flavivirus genus. The immune response to ZIKV was the most distinct, showing rapid B cell expansion but attenuated/delayed B cell activation.

**Conclusion:**

These findings reveal that early immune responses to arboviruses are defined more by infection-specific dynamics than by viral taxonomy, underscoring distinct immunological signatures for each virus.

## Highlights

- Our findings highlight the distinct and nuanced immune responses elicited by CHIKV, DENV, and ZIKV infections in pediatric population.
- CHIKV infection triggers a strong innate and adaptive immune response with significant monocyte and NK cell involvement, transitioning to a robust humoral response during recovery from infection.
- DENV infection prompts a prominent T cell-mediated response with sustained activation.
- ZIKV elicits a more subdued innate response but shows a unique adaptive profile with rapid expansion of the B-cell compartment but delayed B cell activation.

## Introduction

The four dengue virus serotypes (DENV1-4), Zika virus (ZIKV), and chikungunya virus (CHIKV) are widely distributed arthropod-borne viruses (arboviruses). CHIKV and DENV are endemic in tropical and subtropical regions and pose an ongoing threat to public health and socioeconomic development in these areas (1, 2). ZIKV was a major public health concern in 2016, with 89 countries reporting autochthonous mosquito-borne transmission of ZIKV, including those in the Pacific Islands, Central and South America, and the Caribbean, and has recently shown some resurgence in India, with sporadic cases reported in Latin America (3, 4). More recently, all three viruses have shown concurrent circulation in Gabon (2021), Thailand (2018-2020), and Colombia (2019-2020) (5–7). The massive global burden of these viruses necessitates the development of effective vaccines and anti-viral treatments; however, despite extensive research, there are only two licensed DENV vaccines (with one discontinued), one for chikungunya, and no vaccines available for ZIKV. Additionally, another dengue vaccine completed phase-3 clinical trials and is awaiting regulatory decision. Although these viruses are all transmitted by *Aedes* mosquitos, CHIKV is an alphavirus (*Togaviridae* family), whilst DENV and ZIKV are flaviviruses (*Flaviviridae* family) (8). Developing safe vaccines for DENV and ZIKV is particularly challenging because of immunological cross-reactivity between these related viruses. Cross-reactivity among DENV serotypes (DENV1-4) also cause complications for vaccine development and disease severity. For example, secondary DENV infection with a distinct serotype from the primary infection is a known risk factor for more severe disease, attributable in part to antibody-dependent enhancement (9–14). This potential for infection history to influence the severity of subsequent disease has also been seen for Dengvaxia^®^, where vaccine recipients with no prior DENV exposure had a higher risk of hospitalization during a subsequent DENV infection (15–17). Further, prior ZIKV infection has been shown to enhance subsequent dengue severity (18–20). This complex relationship between infection history and disease severity necessitates a deeper understanding of the immune responses to CHIKV, DENV and ZIKV.

All three viruses broadly share commonly observed clinical manifestations, including fever, headache, joint pain (arthralgia), and muscle pain (myalgia); however, they also exhibit distinct clinical profiles, particularly in terms of severe and chronic signs and symptoms (21). The role of T cells in CHIKV, DENV, and ZIKV infections was reviewed recently (22), where it was noted that despite many studies on the role of T cells in CHIKV infection, ‘elucidation of the exact function of T cells is hampered by absence of comprehensive human studies’. In contrast, the role of T cells in DENV infection has been studied in more detail but is still on-going. ZIKV stands out with most patients being asymptomatic, but with potential to cause serious complications in pregnant women, including fetal demise and congenital abnormalities (23). Studies examining the role of T cells in ZIKV infection are also limited.

Here we present a comprehensive, multi-omic study comparing human immunological and inflammatory responses during infection with CHIKV, DENV, or ZIKV, providing greater insight into immune mechanisms underlying the clinical profiles of each virus, with a focus on T-cell, B-cell and NK-cell subpopulations. The patient samples in this study were analyzed as part of the Dengue Human Immunology Project Consortium (DHIPC), which established a program to study DENV using well-characterized samples from children in ongoing clinical studies in Managua, Nicaragua. However, during the study period, major epidemics of chikungunya (2014-2015) and Zika (2016) occurred; thus, samples from children infected with these viruses were also analyzed. These data therefore presented a unique opportunity to compare the immune responses to these three viruses.

We performed immune cell profiling in each patient using mass-cytometry (CyTOF) to characterize and quantify the immune-cell profiles of each patient, both during acute infection and recovery phase. Additionally, we used multiplexed bead-based assays to measure the concentrations of circulating cytokines, chemokines and other inflammatory signaling molecules. This provided a unique opportunity to examine and compare the immune response at the cellular and cytokine/chemokine/growth-factor level, as well as apply the multi-omic analysis technique DIABLO (‘Data Integration Analysis for Biomarker Discovery using Latent cOmponents’) to unravel the complex interplay between these two systems (24). To our knowledge, this is the first time that these three viruses have been analyzed using a similar patient population and the same experimental protocols and compared within the same study. Thus, this study provides a unique global view of the cellular and soluble immune mediators of three major arboviral infections in children. Characterizing the immune response to these three viruses provides a deeper understanding of the complex relationship between infection mechanism, inflammation, immune response, and symptoms and generates hypotheses that inspire future research studies.

## Results

### Study design and characteristics of participants

This study involved 127 pediatric participants of the Pediatric Dengue Cohort Study (PDCS) or the Pediatric Dengue Hospital-based Study (PDHS) in Managua, Nicaragua (14, 25–27). Samples from a total of 45 CHIKV-, 32 DENV- and 50 ZIKV-infected children were included in this study; the dengue and chikungunya cases were from the PDHS and the Zika cases derived from the PDCS. The 32 dengue cases consisted of 13 children with primary DENV1 infection and 19 participants with primary DENV3 infection. The ZIKV and CHIKV sample sets have been described and analyzed previously (28, 29). Participants included in this study had no history of prior DENV infection. The mean age of the chikungunya cases was 9.0 (Standard Deviation [SD] 4.5) years) and that of the Zika cases was 9.0 (SD 3.1) years, with the DENV patients being somewhat younger (6.6 [3.18] years) (**Table 1**). The Zika cases were 48% male, dengue cases were 50% male, and chikungunya cases had a higher proportion, with 71% male. The imbalance of age and gender between the three viruses was accounted for when fitting statistical models (see Methods). Samples were collected at two time-points: acute (days 1–3 or 4–6 post-symptom onset [p.s.o]), and recovery (days 14–25 p.s.o) (**Table 1**). Due to logistical constraints during the ZIKV epidemic, PBMCs from Zika cases (PDCS) could only be collected and processed at days 4–6 p.s.o.; thus, CyTOF data from ZIKV patients are from later days p.s.o. than CHIKV and DENV. Plasma (DENV, CHIKV) or serum (ZIKV) samples and peripheral blood mononuclear cells (PBMCs) were collected for Luminex and CyTOF, respectively, for both time-points. PBMCs were isolated from whole blood as described previously (28, 30). Sampling times closely adhered to the targeted acute (standard deviation [SD] = 1 days) and recovery (SD = 1.7 days) timepoints, however, the timing of the recovery DENV samples was slightly more varied (SD = 2.5). Symptom data are summarized in **Table 2**. Overall, and as expected, ZIKV patients exhibited the fewest symptoms, with symptoms such as fever, arthralgia, and hemorrhagic manifestations all being less common in ZIKV patients relative to CHIKV and DENV. Retro-orbital pain was more prevalent in DENV patients (44%), whereas arthralgia was most common in CHIKV patients (88%).

**Table 1:** Characteristics of study population.

Characteristic	All	CHIKV	DENV	ZIKV	P-value
Participants	127	45	32	50	
Age (mean (SD))	8.40 (3.8)	9.0 (4.5)	6.56 (3.18)	9.0 (3.1)	0.006
Male	72 (57%)	32 (71.1%)	16 (50%)	24 (48.0%)	0.051
Days from Symptom onset (Acute) mean (SD)	1.38 (1.10)	1.52 (0.51)	2.46 (1.26)	0.59 (0.72)	<0.001
Days from Symptom onset (Recovery) mean (SD)	15.12 (1.88)	14.64 (0.62)	16.10 (2.51)	14.91 (1.98)	0.003

**Table 2:** Symptomatic profile of study cohort.

Symptom/Sign	Overall	CHIKV	DENV	ZIKV	P-value
n*	124	43	32	49	
Fever n (%)	109 (87.9)	43 (100.0)	32 (100.0)	34 (69.4)	<0.001
Headache n (%)	47 (38.8)	13 (31.0)	15 (50.0)	19 (38.8)	0.263
Arthralgia n (%)	61 (49.6)	38 (88.4)	6 (19.4)	17 (34.7)	<0.001
Myalgia n (%)	35 (28.7)	17 (40.5)	10 (32.3)	8 (16.3)	0.035
Rash n (%)	107 (86.3)	43 (100.0)	25 (78.1)	39 (79.6)	0.005
Retro-orbital pain n (%)	28 (22.8)	6 (14.3)	14 (43.8)	8 (16.3)	0.004
Facial flushing n (%)	61 (49.2)	36 (83.7)	20 (62.5)	5 (10.2)	<0.001
Vomiting n (%)	17 (13.7)	9 (20.9)	7 (21.9)	1 (2.0)	0.009
Hemorrhagic Manifestations n (%)	25 (20.2)	6 (14.0)	19 (59.4)	0 (0.0)	<0.001
Minimum Platelets (mean (SD))	204.61 (69.21)	189.56 (51.12)	144.97 (47.12)	256.78 (56.59)	<0.001
Average Platelets (mean (SD))	234.35 (73.94)	205.77 (48.55)	175.10 (45.35)	298.12 (58.28)	<0.001

Table shows the number and percentage of participants displaying each symptom, stratified by virus type. Statistical comparisons across the three virus types were performed using chi-squared test for binary variables and ANOVA for continuous variables. *Data was available for all but one Zika case.

### Infection with CHIKV, ZIKV and DENV leads to diverse changes in major immune-cell compartments

**Figure 1:**
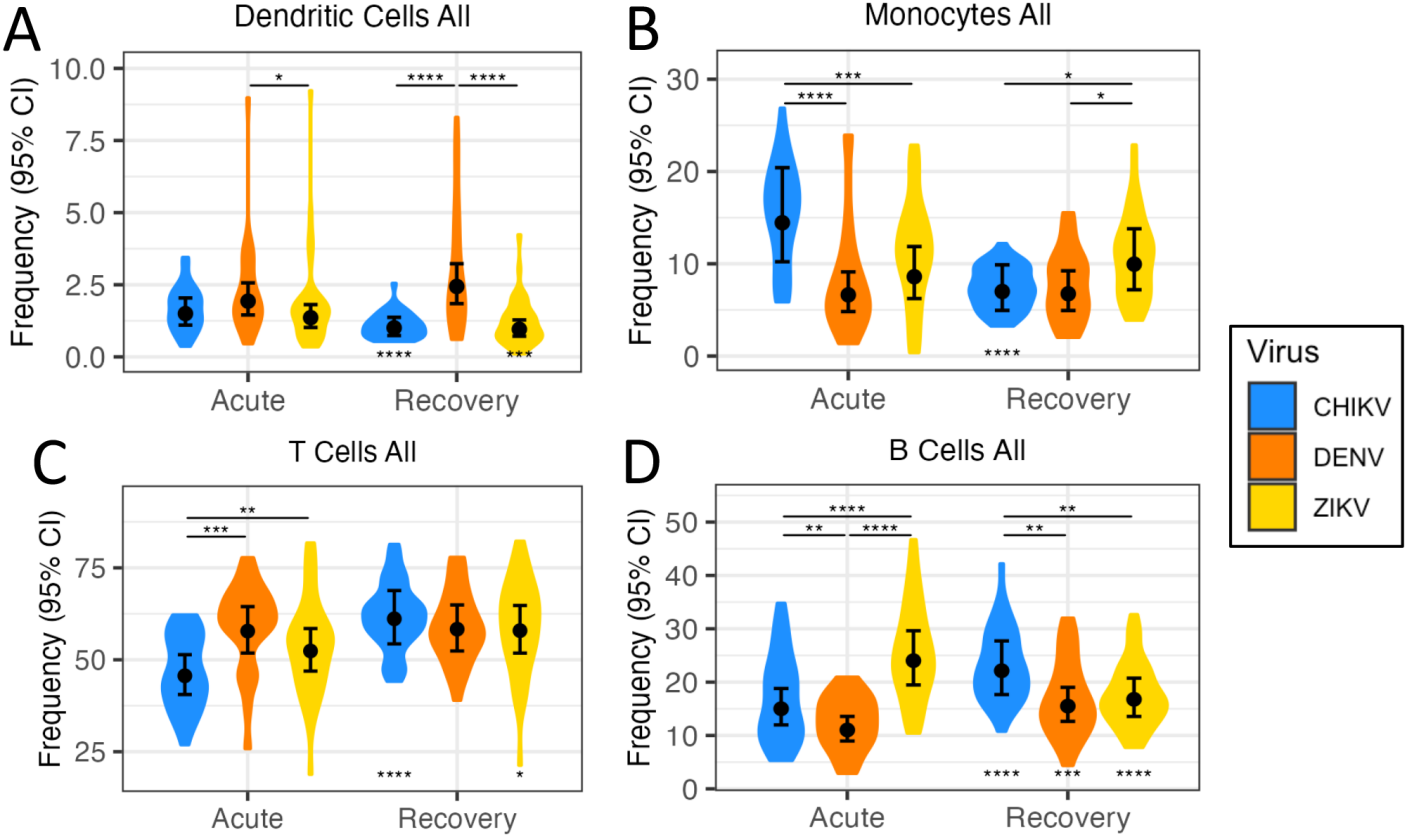
Quantification of immune-cell frequencies following arbovirus infection. Violin plots **(A-D)** show distribution of immune-cell frequencies across patients during acute infection with CHIKV, DENV and ZIKV, as well as during infection recovery. Black circles indicate the mean immune-cell frequency ±95% confidence interval (CI). Asterisks above points indicate significant (FDR<0.05) frequency differences between viruses, whilst asterisks below points indicate significant frequency change from acute infection to recovery. *p<0.05; **p<0.01; ***p<0.001; ****p<0.0001.

We examined and compared the relative frequency of four broad immune-cell compartments (dendritic cells [DC], monocytes, B cells, and T cells) during acute infection and recovery from each virus (**Figure 1**). During acute infection, overall DC frequency was similar for all three virus infections, with ZIKV having the lowest frequency (**Figure 1A**). During infection recovery, DC frequency in chikungunya and Zika cases decreased significantly but remained elevated in dengue cases. A high monocyte frequency during the first days of CHIKV infection (**Figure 1B**) is consistent with a strong innate immune response to CHIKV (29). During infection recovery, monocyte frequency decreased significantly for chikungunya cases, consistent with the ‘switching-off’ of the innate immune response. Interestingly, monocyte frequency in acute DENV and ZIKV infections was lower than acute CHIKV infection and was unchanged from acute to recovery (**Figure 1B**), suggesting that the monocyte response may be muted (modulated) during infection with these two viruses.

The adaptive immune response is thought to begin activation 2–3 days post-infection, peaking ~12 days later (31). Accordingly, we observed differences in T-cell and B-cell frequency during acute infection, reflecting differences in early activation of the adaptive immune response among the viruses. For example, T-cell frequency was much lower than the other viruses during acute CHIKV infection, but increased significantly during recovery, reaching similar levels to the other two viruses (**Figure 1C**). During CHIKV and DENV infection, B-cell frequencies increased significantly from acute to recovery phase, perhaps reflecting the ‘ramping-up’ of the adaptive immune response (**Figure 1D**). However, B-cell frequencies for ZIKV showed highest levels during acute infection, and then decreased significantly during recovery. The later timing of the ZIKV PBMC sample (4–6 days p.s.o) could explain these higher B-cell frequencies.

### Infection with CHIKV, ZIKV and DENV results in distinct concentration profiles of cytokines/chemokines

**Figure 2:**
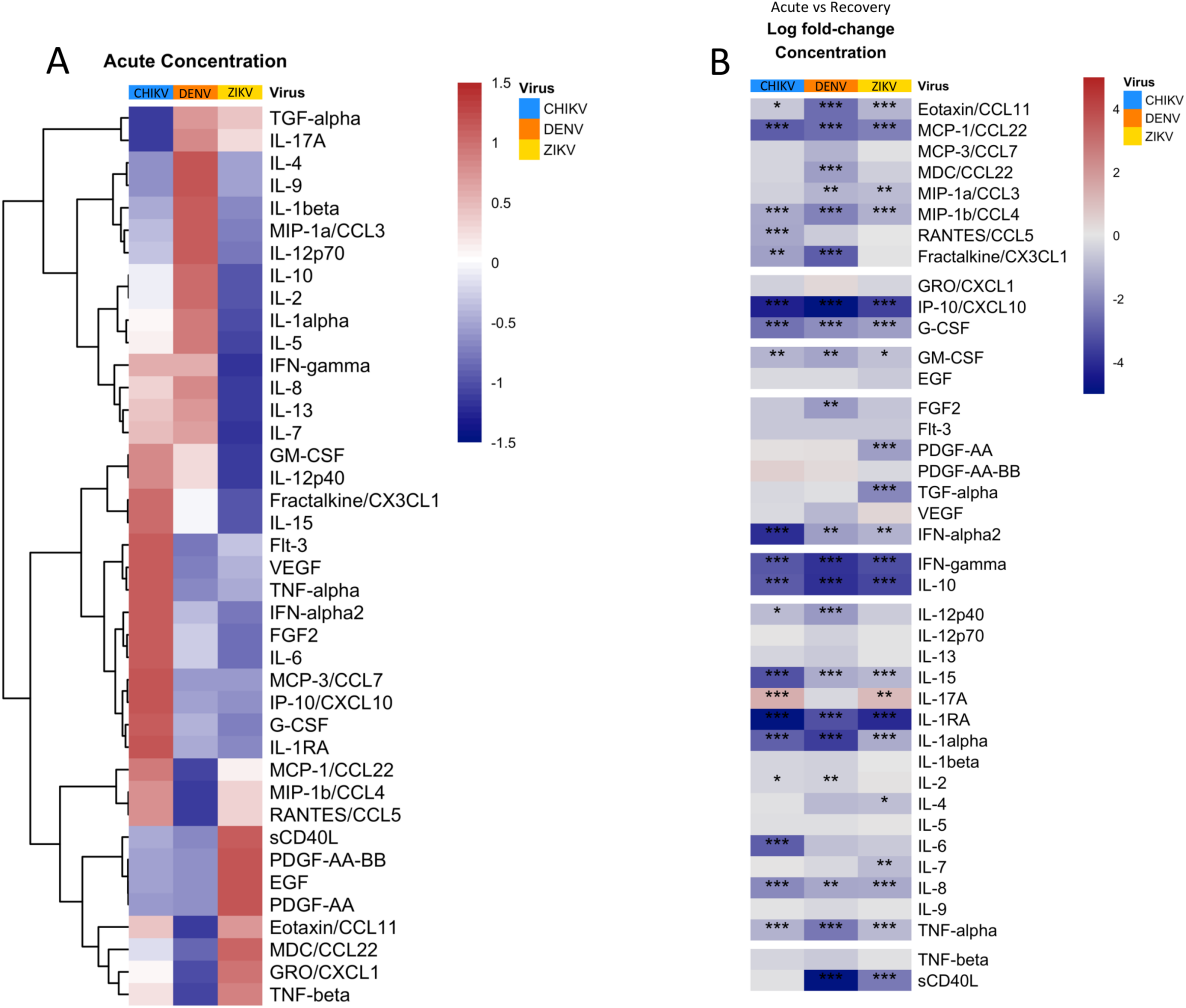
Quantification and clustering of cytokine/chemokine/growth factor concentrations during acute arbovirus infection and transition towards recovery. **(A)** Heatmap showing average concentrations of cytokines/chemokines/growth factors during acute infection, scaled and centered so that 0 (white) is the mean concentration of each protein and 1 (red) and -1 (blue) represent 1 standard deviation (SD) above or below the average, respectively. The heatmap is clustered by row to group proteins with similar concentration profiles. **(B)** Heatmap showing the average log fold-change in protein concentration from acute infection to recovery for each protein. Asterisks indicate significant (FDR < 0.05) concentration change, with *p<0.05; **p<0.01; ***p<0.001; ****p<0.0001. Rows are grouped by ‘protein-type’ as indicated.

We compared the concentration of 40 cytokines, chemokines, and growth factors during acute infection with DENV, ZIKV and CHIKV, revealing significant (FDR<0.05) differences in concentration between viruses (**Figure 2**, **Supplementary Figure S1**). Hierarchical clustering revealed groups with distinct acute cytokine/chemokine response to each virus (**Figure 2A**). Acute infection with CHIKV was characterized by higher concentrations of IP-10/CXCL10, IFN-α, IL-6, and TNF-α, all of which are well-established markers of viral infection and inflammation. Monocyte-chemotactic protein-3 (MCP-3/CCL7) was also highest during CHIKV infection, consistent with the observed high monocyte frequency. Patients infected with CHIKV also showed higher concentrations of the interleukins IL-1RA and IL-15, as well as colony-stimulating factor G-CSF. The cytokine/chemokine response during the innate immune response to ZIKV was distinct from the other viruses due to higher concentrations of the growth factors epidermal growth factor (EGF) and platelet-derived growth factor AA (PDGF-AA), which are known to have anti-inflammatory effects (32–34). Interestingly, average platelet counts were higher in ZIKV patients, consistent with higher PDGF (**Table 2**). ZIKV infection also displayed higher concentrations of sCD40L and macrophage-derived chemokine (MDC). IL-6 concentration was much lower in ZIKV patients relative to CHIKV and DENV, consistent with lower prevalence of fever in ZIKV patients (**Supplementary Figure S1**, **Table 2**). The cytokine profile of acute DENV infection was characterized by significantly higher concentrations of IL-1β, IL-2, IL-4, and IL-9. During infection recovery, the concentrations of most cytokines/chemokines/growth-factors decreased to undetectable for all three viruses (**Figure 2B**). The one exception to this was IL-17, which increased significantly during recovery from CHIKV and ZIKV infection, suggesting activation of the Th17 axis, which is usually associated with defense against extracellular bacterial infections (35). IL-17 is associated with CHIKV-induced arthritic inflammation but it is unclear if IL-17 plays a role in congenial malformation associated with ZIKV (36–39).

### Infection with CHIKV, ZIKV and DENV leads to diverse changes in DC subsets

**Figure 3:**
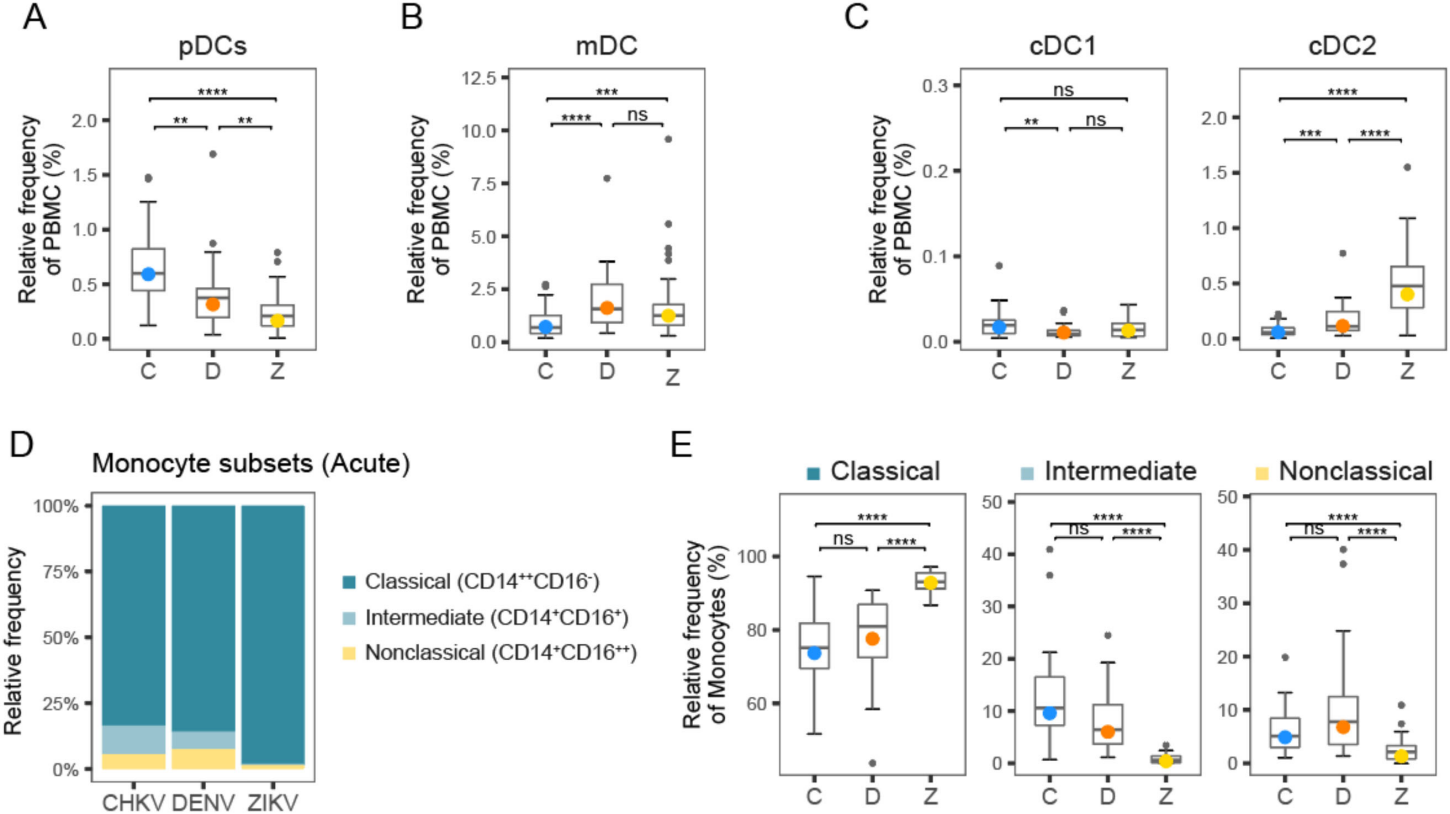
Frequency of DC and monocyte subtypes following arbovirus infection. **(A-C)** Box plots show distribution of DC subtype frequencies across patients during acute infection with CHIKV (C), DENV (D), and ZIKV (Z). **(D)** Stacked bar plot shows relative proportions of classical, intermediate, and non-classical monocytes during acute infection with CHIKV, DENV and ZIKV. **(E)** Box plots show distribution of monocyte subtype frequencies across patients during acute infection with CHIKV, DENV and ZIKV. Colored circles, as in **(A-C, E)**, indicate the mean frequency; asterisks indicate significant (FDR<0.05) differences between viruses. *p<0.05; **p<0.01; ***p<0.001; ****p<0.0001. ns, not significant.

Examination of major DC subtypes revealed differences in acute DC composition among viruses (**Figure 3**). Plasmacytoid DC (pDC) frequency during acute infection followed a clear hierarchy with CHIKV > DENV > ZIKV (**Figure 3A**). The elevated proportion of DCs in DENV patients appears to be primarily driven by an increased frequency of myeloid DCs (mDC (**Figure 3B**). mDCs are a major infection target for DENV, and thus DENV might be expected to affect DCs differently from the other viruses (40). There was little difference in the proportion of classic type 1 DC (cDC1) among viruses (**Figure 3C**); however, classic type 2 DCs (cDC2) proportions were much higher in ZIKV patients (**Figure 3D**).

### Relative frequency of intermediate and non-classical monocytes is highest during acute CHIKV and DENV infection, relative to ZIKV

Circulating monocytes are considered a heterogeneous and dynamic cell population, comprising multiple subsets of monocytes. A higher frequency of total monocytes during acute CHIKV infection compared to DENV and ZIKV may indicate a stronger innate immune response to CHIKV (**Figure 1B**). Closer examination of the monocyte subset frequencies during acute infection revealed that acute infection with CHIKV and DENV showed higher frequency of intermediate (5-10%) and non-classical (~5%) monocytes relative to ZIKV infection (<5%), suggesting higher innate immune activity (**Figures 3D, E**). These observations are consistent with our previous observations of significantly higher frequency of CD16^+^ monocytes during DENV infection relative to ZIKV (41). Additionally, we have demonstrated high frequency of intermediate monocytes during CHIKV infection; here, we observe that they are also elevated during DENV infection, albeit to a lesser extent (41).

### CHIKV, DENV and ZIKV infections lead to distinct, but overlapping, NK cell profiles in acute phase of infection

**Figure 4:**
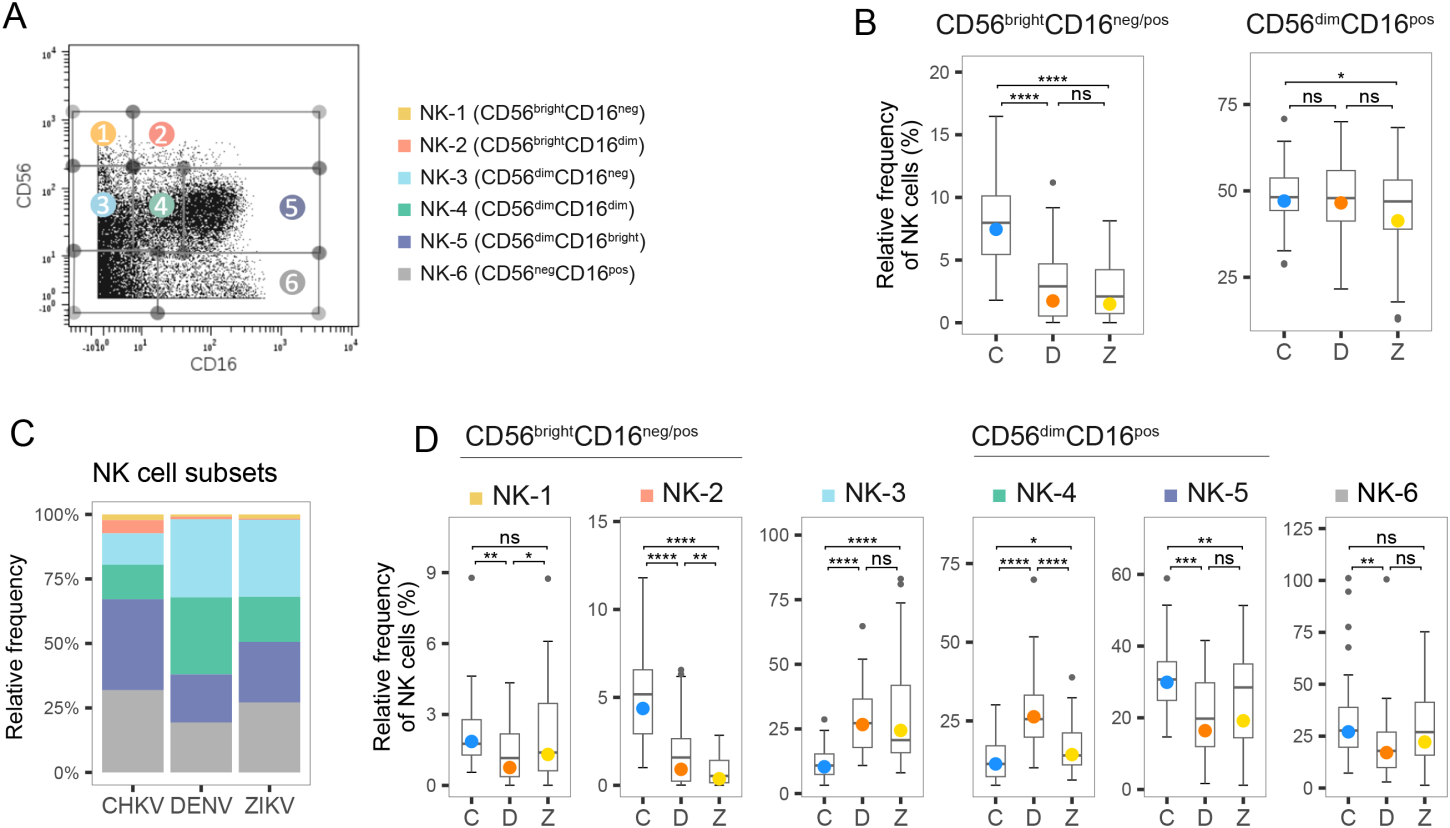
Frequency of NK cell subtypes during acute infection with CHIKV, DENV and ZIKV. **(A)** Scatter plot for a representative PBMC sample shows fluorescence of CD16 and CD56 measured by mass-cytometry. NK cell subtypes 1–6 are indicated. **(B)** Box plot shows distribution of the two canonical compartments within total NK cells: CD56brightCD16neg/pos (left) and CD56dimCD16pos (right) during acute infection with CHIKV (C), DENV (D), and ZIKV (Z). **(C)** Stacked bar plot shows the percentage of NK cell subtypes within the NK cell compartment during acute infection with CHIKV, DENV and ZIKV. **(D)** Box plots show the percentage of NK cell subtypes within the NK cell compartment during acute infection with CHIKV, DENV and ZIKV. Colored circles, as in B and D, indicate the mean frequency; asterisks indicate significant (FDR<0.05) differences between viruses. *p<0.05; **p<0.01; ***p<0.001; ****p<0.0001. ns, not significant.

We measured CD16/CD56 expression to dissect the NK cell compartment, after gating out CD3+ T cells, CD19+ B cells and CD14+ monocytes. We identified the canonical NK subsets CD56^bright^CD16^neg/pos^ and CD56^dim^CD16^pos^, and six NK subpopulations: CD56^bright^CD16^neg^(NK-1), CD56^bright^CD16^dim/bright^ (NK-2), CD56^dim^CD16^neg^ (NK-3), CD56^dim^CD16^bright^ (NK-5), and CD56^neg^CD16^bright^ (NK-6), as well as an intermediate subset CD56^dim^CD16^dim^ (NK-4) that was described by Amand et al. (42) (**Figure 4A**). As expected, most NK cells were found in the CD56^dim^ compartment (**Figure 4B**, indicating that rapid maturation of circulating NKs is a shared hallmark of acute arboviral infection. However, the distribution of circulating NK cell subsets was altered in a virus-dependent manner (**Figures 4B-D**). Participants infected with CHIKV had a higher percentage of CD56^bright^ NK cells, which are known for their immunomodulatory role, with a three-fold higher percentage than observed in DENV or ZIKV infection (**Figure 4B**). CHIKV infection was associated with a cytotoxic response strongly skewed towards antibody-dependent cellular cytotoxicity, with a proportion of CD56^dim^CD16^bright^ (NK-5) two-fold greater than that observed during flavivirus infection (40% vs. 20% in DENV and ZIKV infection) (**Figure 4D**). In contrast, the majority of CD56^dim^ NK cells in ZIKV-infected patients were CD56^dim^CD16^neg^ (NK-3), indicative of recent activation and degranulation (**Figure 4D**). While a similar proportion of CD56^dim^CD16^neg^ was observed after both flavivirus infections, acute dengue was characterized by a marked overrepresentation of the intermediate subset CD56^dim^CD16^dim^ (NK-4, 30%), and the lowest percentage of CD56^bright^CD16^neg^ (NK-1) NK cells (**Figure 4D**). Of note, CD56^neg^CD16^bright^ (NK-6) cells accounted for roughly one-third of the circulating NK pool in all three infections and were significantly higher in CHIKV than DENV (median 35% vs 20%). Because NK-6 cells are highly FcγRIII-driven and typically arise under sustained Fc engagement, their enrichment further supports an antibody-skewed innate response unique to CHIKV (43).

We then analyzed the proportion of NK cells expressing CD57, a maker of terminal differentiation whose frequency is known to rise with age (**Supplementary Figure S2**) (44). CHIKV-infected children (mean age 9 years) displayed the highest frequency of CD57^pos^ NK cells in acute phase, mirroring the rapid and robust induction of CD57 reported in adult population (45). ZIKV-infected children, who were similar in age to the CHIKV infection group (mean age 9 years), showed a significantly lower frequency of CD57^pos^ NK cells frequency, whereas the younger DENV cohort (mean age 6.8 years) exhibited the lowest levels. While the frequency of CD16^pos^ NK cells was comparable between the two flaviviral infection groups (**Figure 4B**), a significantly higher proportion of CD57^pos^ NK cells was observed in ZIKV-infected individuals compared to those infected with DENV. Moreover, the number of CD57 ^pos^ NK cells increased between acute and recovery phase of Zika (but not in CHIKV and DENV), suggesting slower NK cell maturation kinetics. Together, these data show that although age contributes to baseline CD57 expression, the magnitude and kinetics of CD57 induction display different patterns depending on the infecting virus.

### Infection with CHIKV, DENV or ZIKV results in distinct T-cell responses, including highest T-cell activation in DENV

**Figure 5:**
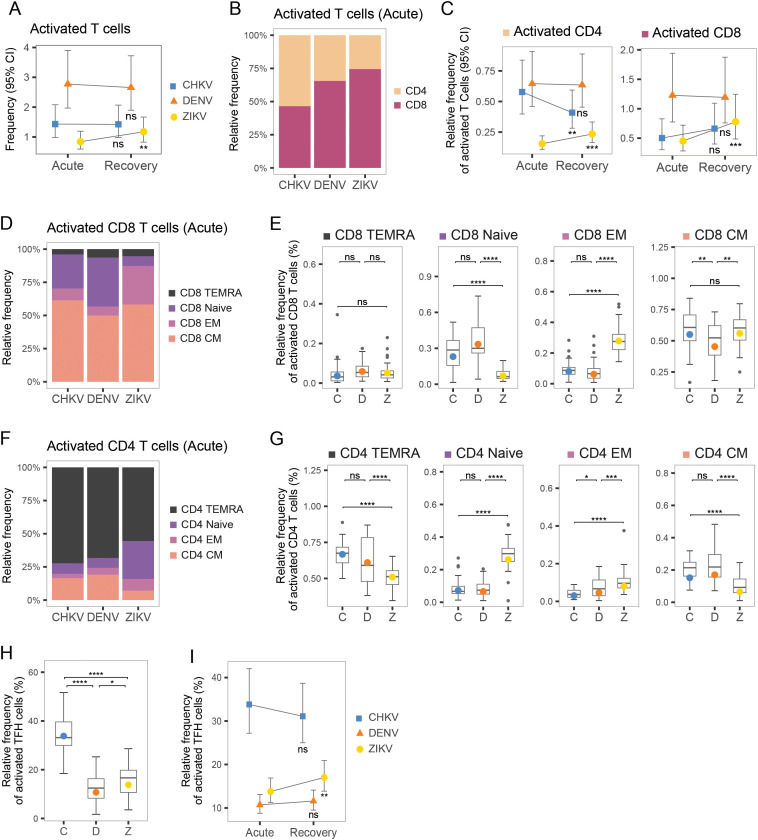
T-cell composition during acute infection with CHIKV, DENV and ZIKV and recovery. **(A)** Scatter plots show mean frequency (+95% CI) of activated T cells within the T-cell compartment during acute infection and recovery. **(B)** Bar plot shows proportions of CD4+ and CD8+ T cells within the activated T-cell compartment. **(C)** Scatter plots show mean frequency (+95% CI) of activated CD4+ and activated CD8+ cells, within the activated T-cell compartment, during acute infection and recovery. **(D)** Stacked bar plot shows relative proportions of Naïve, TEMRA, EM and CM cells within the activated CD8+ T-cell compartment. **(E)** Box plot shows the distribution of Naïve, TEMRA, EM and CM CD8+ T cells within the activated CD8+ T-cell compartment during acute infection with CHIKV (C), DENV (D), or ZIKV (Z). **(F)** Stacked bar plot shows relative proportions of Naïve, TEMRA, EM and CM cells within the active CD4+ T-cell compartment. **(G)** Box plot shows the distribution of Naïve, TEMRA, EM and CM CD8+ T cells within the activated CD4+ T-cell compartment, during acute infection with CHIKV (C), DENV (D), or ZIKV (Z). **(H)** Box plots show relative frequency of activated T-follicular-helper (Tfh) cells (% of helper-cell compartment) during acute infection with each virus. **(I)** Scatter plots show relative frequency of activated Tfh cells (% of helper-cell compartment) during acute infection and recovery for each virus. Colored symbols, as in **(A, C, I)**, indicate the mean frequency; asterisks indicate significant (FDR<0.05) frequency change from acute infection to recovery **(A, C, I)** or significant differences between viruses **(E, G, F)**. *p<0.05; **p<0.01; ***p<0.001; ****p<0.0001. ns, not significant.

Overall, T cells were the most abundant of the cell types measured, accounting for >50% of total PBMCs during infection recovery (**Figure 1C**). The frequency of activated T cells (CD38^+^ HLA-DR^+^) was highest during DENV infection (>2% of T cells) (**Figure 5A**). T-cell activation appeared to be sustained from acute to recovery phases in CHIKV and DENV infection (**Figure 5A**). When examining the relative proportions of CD4^+^ and CD8^+^ in the activated T-cell compartment, acute DENV and ZIKV infection were predominantly CD8^+^ (**Figures 5B, C**). In comparison, acute CHIKV infections showed the highest proportion of CD4^+^ cells, as well as activated CD4^+^ Tfh cells (**Figures 5B, H, I**), suggesting a stronger focus on activation of long-term, antibody-dependent, adaptive immunity via B-cell activation. This is in further agreement with the hypothesis that the innate response to CHIKV clears viremia early, and thus fewer cytotoxic T cells are needed to kill infected cells. Within the active CD8^+^ compartment, central memory (CM) T cells were the most activated, with acute CHIKV and ZIKV infection showing slightly higher activated CM (~55%) than DENV infection (~45% activated) (**Figures 5D, E**). In comparison, the activated CD4^+^ compartment was predominantly composed of Terminal effector memory T cells re-expressing CD45RA (TEMRA) cells, although the proportion of TEMRA cells within the active CD4^+^ compartment was significantly higher during acute CHIKV and DENV infection compared to ZIKV (**Figures 5F, G**). Instead, ZIKV cases exhibited a significantly larger contribution of EM cells in the activated CD4^+^ compartment.

### CHIKV infection shows increased frequency of plasma B cells during CHIKV recovery

**Figure 6:**
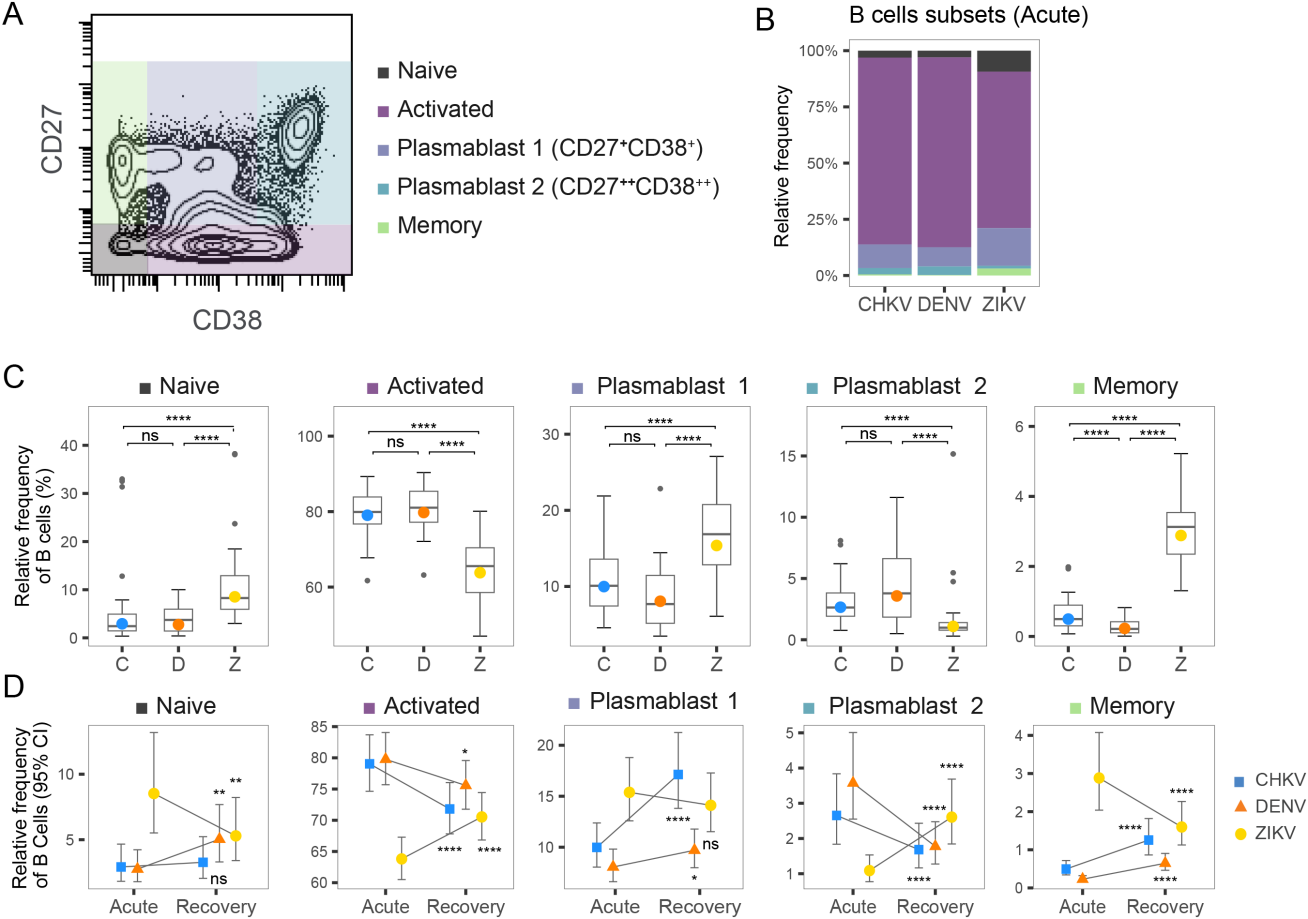
Frequency of B-cell subtypes following arbovirus infection. **(A)** Contour plot of distribution of CD27 and CD38 CyTOF signal across a representative PBMC sample. Colored regions indicate Naïve, Activated, Plasma 1, Plasma 2 and Memory B cells. **(B)** Stacked bar graph shows relative frequency of B-cell subtypes within the activated B-cell compartment during acute infection with CHIKV, DENV, or ZIKV. **(C)** Box plot shows the distribution of indicated Naïve, Activated, Plasma 1, Plasma 2 and Memory B cells within the active B-cell compartment during acute infection with CHIKV **(C)**, DENV (D), or ZIKV (Z). Colored circles indicate the mean frequency; asterisks indicate significant (FDR<0.05) differences between viruses. **(D)** Scatter plots show mean frequency (+95% CI) of B-cell subtypes within the B-cell compartment during acute infection and recovery. Asterisks indicate significant (FDR<0.05) frequency change from acute infection to recovery. *p<0.05; **p<0.01; ***p<0.001; ****p<0.0001. ns, not significant.

We examined the composition of the B-cell compartment following acute infection with CHIKV, DENV and ZIKV, as well as during infection recovery. We identified five sub-populations of B cells – naïve, activated, memory, and two types of plasmablasts, Plasma 1 (CD27^+^ CD38^+^) and Plasma 2 (CD27^+^ CD38^+^) – based on the abundance of CD27 and CD38 surface markers (**Figures 6A, B**). During acute infection, activated B cells were the largest compartment for all three viruses, followed by Plasma 1 cells (**Figure 6B**). B-cell composition was similar during acute CHIKV and DENV infection, both showing higher B-cell activation and Plasma 2 composition than ZIKV (**Figure 6C**). In contrast, acute ZIKV infection was characterized by lower B-cell activation and higher proportions of naïve B-cells, Plasma 1 cells, and memory B cells. Examining the changes in B-cell composition from acute infection to recovery revealed some interesting similarities and differences among the viruses (**Figure 6D**). Transition to recovery was similar for CHIKV and DENV, both of which showed a decrease in activated B cells, a decrease in Plasma 2 cells, and an increase in memory B cells. Despite these similarities, we observed a large increase in the Plasma 1 compartment (10%-17%) during recovery from CHIKV that was not observed for DENV. In contrast, recovery from ZIKV showed an increase in activated B cells and a decrease in naïve and memory B cells, perhaps indicative of a delayed or attenuated B-cell response.

### Multi-omic analysis identifies immune components that differentiate the acute immune responses to CHIKV, DENV and ZIKV

To achieve a deeper understanding of the interplay between cell-type abundance and cytokine/chemokine/growth-factors profiles, we conducted a multi-omic analysis using Data Integration Analysis for Biomarker discovery using Latent cOmponents (DIABLO). DIABLO is a multi-omic analysis tool that identifies combinations of ‘features’ from different omics platforms (e.g., proteomics, CyTOF) that best explain the differences between sample groups. We utilized DIABLO to identify the combinations of cytokines/chemokine and immune cell types that best characterize the immune response to each virus, providing a more integrated understanding of the complex relationship between viral infection, inflammatory signaling and immune-cell behavior.

**Figure 7:**
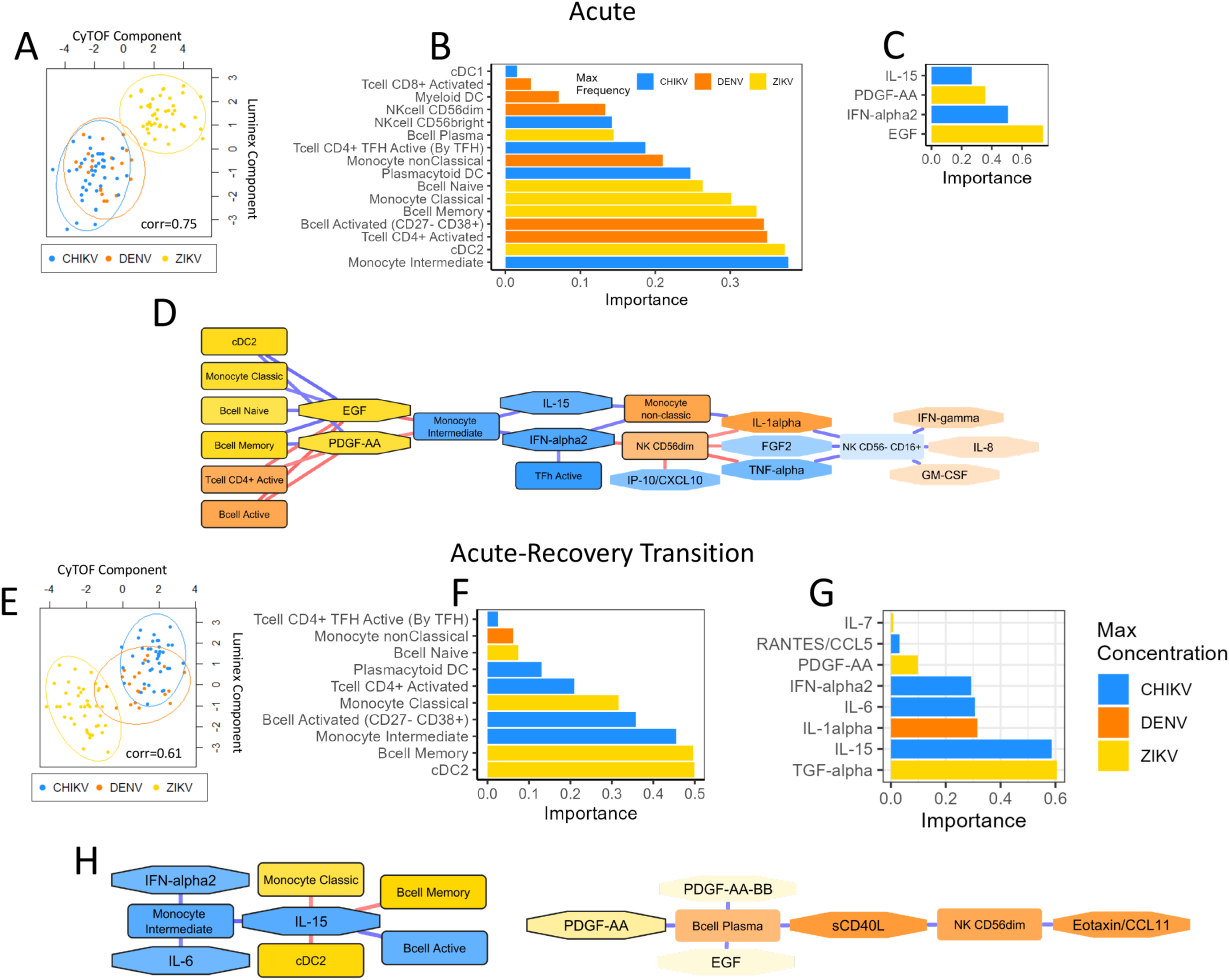
Multi-omic analysis of cytokine/chemokine/growth factors and immune-cell profiles during acute infection and acute-recovery transition. Plots summarize the multi-omic DIABLO analysis of inflammatory proteins and immune-cell profiles during acute infection **(A-D)** and the acute-recovery transition **(E, F)**. Scatter plots **(A, E)** show Luminex and CyTOF component scores for each sample. Samples that are closer together have more similar Luminex and CyTOF profiles. Data points are colored by virus type for each sample. The correlation score indicates the Pearson’s correlation between each component. Bar plots **(B, C, F, G)** show the weights (importance) of each feature in the DIABLO analysis; these ‘important’ features explain most of the variation between each virus type. The color of each bar indicates the virus showing the highest expression of that feature. Network diagrams **(D, H)** show Pearson’s correlation between each feature (nodes). The strength and direction of the correlation is indicated by the color of each edge (blue=up, red=down). Only features with correlation >0.7 are shown. The color of each node indicates the virus that showed highest expression of that feature. ‘Important’ features are highlighted with a black boarder.

We initially focused our multi-omic analysis on the acute immune response and included all monocyte and NK subtypes, DC subtypes, activated T-cell subpopulations, and B-cell subtypes, as well as all cytokines/chemokines. This analysis revealed a strong correlation (r=0.75) between cytokine/chemokine protein concentrations and immune-cell frequencies, indicating a strong relationship between these immune components during the acute response (**Figure 7A**). This analysis reveals that chikungunya and dengue cases were more similar in terms of immune-cell and inflammatory profiles, whilst Zika cases had a more distinct immune response (**Figure 7A**). The absolute value of the feature weighting represents the ‘importance’ of that feature for differentiating between the immune responses to each virus (**Figures 7B, C**). Intermediate monocytes were the most important cell type for differentiating the immune responses to each virus, showing highest frequency during acute CHIKV infection. cDC2 and activated CD4^+^ T-cells were the second and third most important differentiators, showing highest frequency during ZIKV and DENV infection, respectively. EGF and IFN-α2 were the two most important inflammatory proteins, with EGF concentration being highest during ZIKV infection and IFN-α2 being highest in CHIKV. The key conclusion of this analysis is that the acute immune response to CHIKV and DENV are more similar, characterized by high IFN-α, intermediate monocytes, and CD4 activation, whilst the ZIKV response is more distinct from these, characterized by high levels of EGF and high levels of cDC2.

A network diagram based on the DIABLO analysis shows immune-cell types and inflammatory proteins with high correlation (**Figure 7D**). Notable network features include intermediate monocytes as a hub-node negatively correlated with EGF and PDGF-AA and positively correlated with IL-15 and IFN-α2. It is notable that intermediate monocytes, IL-15 and IFN-α are all important differentiators for the immune responses to each virus, showing highest frequency/concentration during acute CHIKV infection, suggesting that these three features strongly characterize the CHIKV immune response. Similarly, EGF and PDGF-AA form a hub that positively correlated with frequency of cDC2, classical monocytes, naïve B cells and memory B cells, characterizing the immune response to ZIKV. In contrast, the frequency of activated CD4^+^ T cells and activated B cells characterized the DENV immune response and were negatively correlated with EGF/PDGF-AA. Overall, the DIABLO analysis highlights key characteristics of each viral infection response and reveals interesting relationships between immune features. This network diagram provides an overall picture of the inflammatory mediators and immune-cells that characterize the responses to CHIKV, DENV and ZIKV, and highlights the associations between them.

### Multi-omic analysis identifies immune components that differentiate the acute-recovery transition for CHIKV, DENV and ZIKV infection

We then used DIABLO analysis to examine the acute-recovery transition, based on the same immune components as the acute analysis. The PCA plot for this analysis revealed greater overlap in immune-cell and inflammatory protein profiles for each virus, with DENV profiles overlapping CHIKV (**Figure 7E**). The correlation of immune-cell and cytokine/chemokine/growth-factors profiles (Pearson’s r=0.61) was lower for the ‘acute-recovery’ transition compared to the acute infection, suggesting some disconnect between immune-cell frequency and inflammatory proteins during the recovery phase. This could suggest that cytokines/chemokines proteins play a more important role during the acute stage of infection but become less important during infection recovery. Recovery from ZIKV infection showed the biggest decrease in cDC2 and memory B cells, as well as the biggest decrease in TGF-α (**Figures 7F, G**), relative to the other viruses. Recovery from CHIKV infection was characterized by the greatest decrease in intermediate monocytes and activated B cells, whilst a decrease in IL-1α concentration was most characteristic of DENV recovery. The network diagram from the recovery transition analysis revealed 2 distinct clusters of immune components (**Figure 7H**). The largest cluster was comprised of 8 nodes, with IL-15 acting as a hub positively correlated with intermediate monocytes and activated B cells, indicating that recovery from CHIKV is characterized by large decreases in these immune features. The second cluster showed that decreases in PDGF-AA during recovery coincide with decreases in plasma B cells, sCD40L, NK CD56^dim^, and eotaxin. The key conclusion of this analysis is that changes in inflammatory profiles and immune cells when recovering from CHIKV and DENV are similar to each other, whereas recovery from ZIKV is more distinct.

## Discussion

Our study provides valuable insights into the distinct immune responses elicited by infection with the alphavirus CHIKV and the flaviviruses DENV and ZIKV, revealing significant differences in both the innate and adaptive immune compartments. Interestingly, our DIABLO analysis revealed that these immune-cell and inflammatory profiles did not cluster according to viral genus (Alphaviridae vs. Flaviviridae); rather, CHIKV and DENV cases exhibited greater similarity to each other, while ZIKV cases displayed a more distinct immune signature. By comparing immune cell frequencies and cytokine concentrations during acute infection and the transition to recovery, we identified unique immune strategies employed by each virus. These findings offer insights into pathogenesis and immune response to these viruses.

### Innate immune response

The innate immune response plays a crucial role in the initial defense against viral infections. DCs, sentinels of the immune system, play a pivotal role in the initiation of adaptive immune responses via cytokine production and antigen presentation, and they are among the first cell types to be infected upon a mosquito bite. Our findings show that CHIKV infection is associated with higher frequencies of pDCs compared to DENV and ZIKV, consistent with the strong production of IFN-α and activation of NK cells observed. This pattern is concordant with human and mechanistic studies showing that pDC sensing rapidly drives IRF7/type-I IFN programs during CHIKV and can potentiate downstream NK IFN-γ responses (46–48). This suggests that the host’s immune system is mounting a robust antiviral response to control viral spread. In contrast, flavivirus infections showed higher mDC frequencies, consistent with heightened DC participation. Prior work indicates that flaviviruses can infect or modulate human moDCs; notably, ZIKV can suppress NF-kB–driven activation/maturation and reduce T-cell stimulatory capacity, whereas DENV tends to induce pro-inflammatory DC programs (49–51). Consistent with these reports, our results indicate that although mDC frequency increases in both flavivirus infections, ZIKV-exposed mDCs appear numerically expanded yet functionally restrained, whereas DENV-exposed mDCs are both more abundant and functionally activated, in line with the stronger T-cell/IFN-γ signatures we observe.

The innate immune response to CHIKV infection was monocyte-centric, characterized by robust engagement of monocytes, particularly intermediate monocytes. Independent patient cohorts likewise report preferential activation of CD16^+^ monocytes in acute chikungunya with enrichment of TLR7^+^ intermediates/non-classicals monocyte, increased TLR4 on non-classicals monocyte, and elevated soluble CD163, supporting sustained monocyte/macrophage activation. These patterns occur with acute surges of monocyte-recruiting/activating chemokines (IP-10, MCP-1), also observed in our study, and with direct CHIKV infection of circulating monocytes, supporting efficient mobilization and activation of the monocyte compartment during acute disease (48, 52). These signatures are consistent with the strong symptomatic profile associated with CHIKV infection (46, 53, 54). Similarly, DENV infection also showed elevated frequencies of intermediate monocytes, though to a lesser extent, in line with a previous report showing expansion of CD14^+^CD16^+^ monocytes during acute dengue and their role in promoting plasma blast differentiation and anti-DENV antibody responses (55). In contrast, ZIKV infection is associated with a surprisingly high frequency of classical monocytes. In an independent acute ZIKV cohort, CD14^+^ monocytes were relatively preserved, with elevated IP-10/MCP-1 (56), and experimental studies show ZIKV tropism for CD14^+^ blood monocytes and monocyte-derived macrophages (57, 58), all pointing to a predominant involvement of classical monocytes.

Our examination of NK cells further highlights virus-specific immune strategies. CHIKV-infected participants exhibited elevated levels of CD56^bright^ NK cells, along with a higher percentage of CD56^dim^CD16^bright^ (NK-5) NK cells, consistent with a cytotoxic/ADCC-skewed phenotype and the high IFN-γ we observed. Consistent with these observations, acute human CHIKV cohorts show early, transient remodeling of NK phenotype and function, with polarization toward cytotoxicity (59). On the other hand, DENV infection was characterized by a marked overrepresentation of the intermediate CD56^dim^CD16^dim^ NK-4 cells and a lower proportion of CD56^bright^ NK cells, indicating a different mode of immune modulation. This suggests a balanced NK response that combines moderate cytotoxic activity with limited cytokine production, which might help to control the virus while avoiding excessive inflammation. Human dengue cohorts demonstrated early NK activation with tissue-homing imprints and type-I-IFN–dependent NK degranulation against DENV-infected dendritic cells, aligning with a balanced NK response that tempers cytokine production while retaining effector capacity (60). Of note, despite the lower frequencies of CD56^bright^ NK cells in DENV infection, high levels of IFN-γ are observed, indicating that other immune cells, particularly CD8+ T cells, may also play a substantial role in producing IFN-γ in DENV infection (61). ZIKV infection predominantly featured CD56^dim^CD16^neg^ NK cells (NK-3), known for their degranulation capacity, reflecting a strategy focused on direct killing of infected cells. In both DENV and ZIKV, the low frequency of CD56^bright^ NK cells suggests curtailed inflammatory cytokines. Additionally, ZIKV infection displayed low levels of IFN-γ, consistent with a strategic modulation of the immune response by ZIKV to evade excessive inflammation and immune-mediated damage. Mechanistically, ZIKV can upregulate MHC class I on infected cells, dampening NK activation and killing; together with functional restraint despite the presence of degranulation-competent subsets, this provides a parsimonious explanation for muted IFN-γ (62).

### Adaptive immune response

T-cell responses further delineate the immune profiles of these infections. In acute CHIKV infection, we observed lower frequencies of activated T cells compared to DENV infection, with a significant increase during recovery. This pattern suggests that an efficient early innate response controls the virus, thereby reducing the need for an immediate robust T-cell response. Notably, other cohorts do report robust CD8^+^ T-cell activation in acute CHIKV, which might reflect cohort- (adult versus pediatric) and timing-dependent kinetics that can differ from ours. The predominance of CD4^+^ T follicular helper (Tfh) cells in CHIKV in our data is compatible with the vigorous CHIKV humoral response reported in humans, including early neutralizing IgG3 and durable neutralization (63, 64). By contrast, DENV showed the highest frequency of activated T cells in the acute phase, particularly CD8^+^ T cells, highlighting a dominant role of T cell-mediated cytotoxicity, in line with studies reporting HLA-DR^+^CD38^+^ CD8^+^ T-cell expansions and strong DENV-specific effector functions in patients (65). In ZIKV, activated T cells were numerically skewed toward CD8^+^ in the acute phase, while the activated CD4^+^ pool was enriched for effector-memory (EM) cells. This pattern is concordant with a previous report showing broad activation of CD4^+^ and CD8^+^ T cells and activation across memory subsets (66).

The B-cell compartment also revealed virus-specific responses. DENV showed high B-cell activation with an expanded plasmablast compartment during acute infection, followed by a shift to memory B cells in recovery, a trajectory consistent with a “plasmablast burst” and its link to inflammatory monocytes and Tfh cells (55, 67). CHIKV infection convalescence in our cohort was marked by a rise in plasma cells CD38+CD27+, compatible with reports of robust neutralizing antibody responses and maturation of CHIKV-specific humoral immunity over recovery (64, 68). ZIKV, by contrast, showed lower acute B-cell activation with higher naïve/memory frequencies and increased activation during recovery, indicative of a delayed or prolonged humoral response. These findings align with studies that report smaller or more variable plasmablast expansions in early acute ZIKV infection and a convalescent strengthening of ZIKV-specific and cross-flavivirus antibodies (69–72).

### Cytokine/chemokine/growth-factor response

The cytokine profiles further delineate the unique immune responses to each virus. In our cohort, acute CHIKV infection was marked by elevated IP-10, IFN-α, IL-6, and TNF-α, indicating a strong pro-inflammatory state. We also observed high MCP-1 and MCP-3, consistent with increased monocyte frequencies, along with strongly elevated IL-1Ra (anti-inflammatory feedback) and IL-15 (supporting NK/T-cell survival and proliferation), indicating concurrent regulatory feedback and lymphocyte support. IL-8 was likewise increased. These features are consistent with prior reports (53, 73, 74). Comparative acute-phase profiling of acute CHIKV and DENV infection also found IL-8 (and IL-4) upregulation in both infections, with CHIKV-specific downregulation of IL-13 and MCP-3 and age-associated differences; ex vivo PBMC infection recapitulated the IL-8/IL-13/MCP-3 pattern (but not IL-4) (75). Notably, that study did not restrict dengue to primary infection, so the higher MCP-3 in CHIKV that we observe may reflect differences in immune status (primary vs secondary infection) and/or age distribution between studies. In DENV infection, higher concentrations of IL-1β, IL-2, IL-4, and IL-9 reflected a mixed pro-inflammatory and Th2-skewed response. In the literature, IL-1β elevations in dengue patients align with inflammasome activation during infection, and meta-analyses/studies show significant IL-4 and IL-9 increases in acute dengue, consistent with our Th2-associated signals (76–78). Compared with CHIKV and DENV, acute ZIKV in our cohort displayed a muted inflammatory cytokine signature with lower IL-1 family, TNF-α, IL-6 and IFN-γ. Consistent with this restrained pattern, ZIKV also showed the lowest level of IFN-α, in line with a prior study showing that ZIKV limits pDC maturation/activation and pDC capacity to produce type-I IFN (79). Instead, ZIKV showed relatively higher EGF/PDGF and sCD40L/MDC, pointing to a milieu enriched for platelet/vascular and tissue-repair signals rather than broad systemic inflammation. A previous report based on adult patients also reported growth-factor–rich profiles (e.g., PDGF, VEGF/FGF) across early-to-late acute phases (38).

During the recovery phase, the concentrations of most immune proteins decreased to near baseline levels across all three viruses, indicating successful resolution of the infection and restoration of immune homeostasis. An exception was the increased levels of IL-17 during recovery from CHIKV and ZIKV, but not DENV, suggesting activation of the Th17 axis. This activation is potentially linked to autoimmune phenomena or ongoing inflammation, particularly in the context of CHIKV-induced arthritic inflammation.

### Limitations

This analysis has several limitations, many of which are due the ‘uncontrolled’ nature of working with natural human infections. Firstly, the sample size of DENV infection was smaller than the other two viruses and consisted of infection with two distinct DENV serotypes (DENV1/DENV3). This made it difficult to disentangle the DENV1 immune response from the DENV3 response, since serotype-specific comparisons would likely be under-powered. Another issue is that the acute Zika PBMC samples were collected later than the other two virus types, thus distorting the comparison with CHIKV and DENV. Similarly, the timing of the acute plasma samples (for cytokine measurements) was different (on average) between the three virus types (**Table 1**) which might account for some differences. Another issue is the ambiguous interpretation of ‘cell proportions’ measurements from the CyTOF data. For example, the lower frequency of naïve T cells during CHIKV infection could be interpreted as a lower number of naïve or a higher number of other cell types. In this example, we assume a combination of both interpretations, with lower numbers of circulating naïve cells (due to migration to other tissues), accompanied by expansion of non-naïve cell types such as effector memory T cell populations; however, the interpretation for other cell types might be more difficult. In addition, virus-driven death of circulating leukocytes might contribute to the immune signatures we report. DENV, ZIKV, and CHIKV infect human myeloid cells, and infection can precipitate caspase-dependent apoptosis (80, 81). In dengue, primary monocytes are permissive to infection, and infection triggers late activation of caspase-1, IL-1β release and pyroptosis (82). For ZIKV, productive infection of myeloid cells and impaired DC activation/IFN responses have been reported, and transient decline in circulating mDCs has been documented in patients (50, 69, 79). For CHIKV, productive monocyte infection occurs and, in patients, very early CD95/Fas-mediated apoptosis of CD4 T cells accompanies marked lymphopenia; CHIKV also engages intrinsic/extrinsic apoptotic programs with potential bystander effects (48, 52, 83). Collectively, these processes can deplete susceptible or highly activated subsets from blood and contribute cell-death-linked mediators (e.g., IL-1 family), such that our PBMC phenotypes (viability-gated) and cytokines reflect the surviving circulating compartment at sampling. Apparent reductions or between-virus differences may therefore reflect a composite of death and tissue redistribution rather than altered differentiation alone. While our study did not quantify apoptosis/pyroptosis directly, we note this as a limitation and propose future work incorporating annexin V/active-caspase readouts (or death-pathway transcriptional programs), absolute leukocyte counts, and tightly time-matched sampling across etiologies to disentangle depletion by death versus cell trafficking.

Finally, our study design and sample availability limited the scope of our study. Complete viral load data were not available for all the participants included in this study; however, prior work from our group in Nicaragua demonstrated virus-specific viremia distributions and clinical correlations across ZIKV, CHIKV, and DENV (84). Direct assessment of Tregs was also not possible due to the lack of FOXP3 to identify canonical CD4^+^FOXP3^+^ Tregs, and TGF-β was not measured. Accordingly, we interpret PBMC phenotypes and cytokines without attributing them to Tregs.

## Conclusion

In summary, our findings highlight the distinct and nuanced immune responses elicited by CHIKV, DENV, and ZIKV infections. CHIKV infection triggers a strong innate and adaptive immune response with significant monocyte and NK cell involvement, transitioning to a robust humoral response during recovery. DENV infection prompts a prominent T cell-mediated response with sustained activation, while ZIKV elicits a more subdued innate response but shows a unique adaptive profile with delayed B-cell activation. These insights provide a deeper understanding of the immune mechanisms underlying these viral infections and can inform targeted therapeutic strategies and vaccine development.

### Future perspectives

This work highlights several important questions. Firstly, how typical is the CHIKV/DENV immune-response relative to other infections (i.e., not arbovirus); is their response typical of viruses with similar symptomatic profiles such as influenza and SARS-CoV2? Certainly, the response to ZIKV seems less typical; are there other viruses that induce a similar response? Overall, this research highlights the distinct and nuanced immune responses elicited by CHIKV, DENV, and ZIKV infections.

## Methods

### Study participants and samples

Information about age and sex of study participants is shown in **Table 1**. *A. Zika cases*: As part of the Pediatric Dengue Cohort Study (PDCS) in Managua, Nicaragua, blood samples collected from 50 DENV-naïve ZIKV-positive children presenting to the study health center (Centro de Salud Sócrates Flores Vivas) in July and August 2016 were included in this study. Samples were collected at three time-points: early acute (days 1–3 post-symptom onset [p.s.o.]), late acute (days 4–6 p.s.o.) and early convalescence/recovery (day 14–21 p.s.o.). During the study period, PDCS cases that exhibited any of four broad clinical profiles suspected of Zika were eligible for inclusion; there were no exclusion criteria. These clinical profiles were: fever and at least two of headache, retro-orbital pain, myalgia, arthralgia, rash, hemorrhagic manifestations, and leukopenia (1997 WHO dengue case definition); fever and at least two of nausea or vomiting, rash, aches and pains, positive tourniquet test, leukopenia, and any dengue warning sign (2009 WHO dengue case definition); undifferentiated fever without evident cause, with or without any other clinical finding; and afebrile rash, with or without any other clinical finding. ZIKV infection was confirmed by real-time RT-PCR in acute-phase blood and/or urine samples performed at the National Virology Laboratory of the Ministry of Health in Managua using either of two triplex assays that simultaneously detect ZIKV, CHIKV and DENV infections: the ZCD assay (85, 86) or the CDC Trioplex assay (87), with additional confirmation by virus isolation in select cases. In addition, seroconversion by ZIKV IgM capture ELISA in paired acute and convalescent sera was tested (88). Confirmed ZIKV-positive cases were classified as DENV-naïve if they entered the cohort study with no detectable anti-DENV antibodies, as measured by DENV inhibition ELISA (iELISA) assay and had no documented DENV infections (symptomatic or inapparent) during their time in the cohort (14, 89). *B. Chikungunya cases*: As part of a study conducted at the National Pediatric Reference Hospital (Hospital Infantil Manuel de Jesús Rivera; HIMJR) in Managua, Nicaragua, blood samples were collected from 43 children who presented with suspected CHIKV infection between September 2015 and April 2016. Samples were collected at acute (1–2 days p.s.o.) and early convalescent (15–17 days p.s.o.) time-points. CHIKV infection was confirmed by real-time RT-PCR (Waggoner et al., 2016). Additional confirmation included IgM capture ELISA and/or a ≥4-fold rise in antibody titers by iELISA between acute and early convalescent samples (90). All participants were screened for DENV infection, and CHIKV/DENV co-infections were excluded. Children with severe clinical presentations were excluded. *C. Dengue cases*: Blood samples were collected from 32 children who presented with suspected dengue to the HIMJR from August 2010 to December 2013. Samples were collected at acute (day 1–4 p.s.o.) and early convalescent (day 13–25 p.s.o.) time-points. DENV infections were laboratory confirmed by nested RT-PCR or real-time RT-PCR and serotyped by RT-PCR (91). Only primary DENV1 or DENV3 infections—immune status defined by an acute-phase iELISA titer <10—were included (92). After the emergence of ZIKV in 2016, participants were screened for ZIKV infection, and primary DENV cases with prior ZIKV exposure were excluded. For all three sample sets, we used plasma samples from early acute and recovery time-points for Luminex and PBMCs at acute (1–4 days p.s.o.; DENV and CHIKV) or late-acute (4–6 days p.s.o.; ZIKV) and convalescent time-points for CyTOF (**Figure 1**). CHIKV, ZIKV and DENV PBMCs and plasma samples were stored at liquid nitrogen or −80°C respectively. CHIKV and ZIKV samples were assayed within 1 year of collection. DENV samples (collected 2010–2013) were stored at −80°C and assayed 4–7 years after collection. All specimens were aliquoted to avoid repeat freeze–thaw and were shipped/handled via cold chain appropriate to sample type.

### Ethics statement

The study protocols for each study were reviewed and approved by the Institutional Review Boards (IRBs) of the University of California, Berkeley, and the Nicaraguan Ministry of Health. Parents or legal guardians of all subjects provided written informed consent, and subjects 6 years of age and older provided verbal assent as approved by the IRBs.

### PBMC preparation

For PBMC preparation (for mass-cytometry), blood samples were collected in Vacutainer tubes (Becton-Dickenson) with EDTA anticoagulant reagent. Upon receipt in the Nicaraguan National Virology Laboratory, 4–5 mL of blood was transferred into a Leucosep tube (Greiner Bio- One) containing 3 mL of Ficoll Histopaque (Sigma) and centrifuged at 500 x g for 20 minutes (min) at room temperature. The PBMC fraction was collected and transferred to a 15-mL conical tube containing 9 mL of PBS with 2% fetal bovine serum (FBS; Denville Scientific) and 1% penicillin/streptomycin (Sigma). Cells were washed three times in this solution by centrifugation at 500 x g for 10 min and resuspended in 10 mL of complete medium. Before the third wash, an aliquot was used to obtain a cell count using a hematology analyzer (Sismex XS-1000i). After the third wash, cells were resuspended at a concentration of 10^7^ cells per ml in freezing medium consisting of 90% FBS and 10% dimethyl sulfoxide, and aliquoted. Cryovials containing the cell suspension were placed in isopropanol containers (Mr. Frosty, Nalgene) at 80°C overnight and transferred to liquid nitrogen for storage.

### Mass-cytometry (CyTOF) sample processing and acquisition

CyTOF measures up to 41 cell surface analytes at single-cell resolution using metal-labeled reagents and inductively coupled plasma mass spectrometry. Cryopreserved PBMC vials were thawed at 37°C until approximately two-thirds of the volume had thawed, then slowly transferred into 10 mL pre-warmed RPMI containing benzonase. Cells were then stained with the Rhodium (Rh)-103 nucleic-acid intercalator added to culture medium and incubated for 20 min at 37°C Cell viability was determined by Rh103 staining. PBMC samples showing <30% viable cell frequency or those with fewer than 50,000 events were excluded from downstream analyses. Paired PBMC samples from each time-point were first barcoded using a CD45 antibody-based barcoding approach (93), and each acute and recovery paired sample was pooled as a single patient sample for subsequent processing to minimize technical variability and potential batch effects. The pooled patient samples were then stained with a previously tested 37-marker CyTOF antibody panel for 30 min on ice. The samples were fixed, permeabilized and barcoded using commercial palladium (Pd) barcoding kits (Fluidigm) and pooled as sets of 20 samples. These pooled samples were then incubated with Iridium (Ir) nucleic acid intercalator (Fluidigm) in freshly diluted 2% formaldehyde. The samples were stored at 4°C in PBS containing 0.1% BSA until acquisition. Immediately prior to CyTOF acquisition, the samples were washed with deionized water (diH20), counted and resuspended in diH20 containing a 1/20 dilution of Equation 4 Element beads (Fluidigm). Following routine auto-tuning, the samples were acquired on a CyTOF2 mass cytometer (Fluidigm) equipped with a SuperSampler fluidics system (Victorian Airships) at an event rate of <400 Hz. Each composite barcoded sample required approximately 20 hours of acquisition time. Manual gating was performed to identify distinct cell populations based on marker expression using the Cytobank version 5.2.0 (Cytobank Inc.) software. (See **Supplementary Table S1** for gating definitions used for each cell type, as well as **Supplementary Methods** for more detailed gating).

### Multiplex ELISA (Luminex)

Cytokines, chemokines, and growth factors were measured using a multiplex bead‐based assay (Luminex). Each sample was run in duplicate in a 96‐well microtiter plate using 25 μl serum from each patient from acute and recovery time-points using multiplex cytokine panels (Multiplex High Sensitivity Human Cytokine Panel, Millipore Corp.). Forty analytes (cytokines and chemokines) were measured using a Luminex‐200 system and the XMap Platform (Luminex Corporation). Acquired mean fluorescence data were analyzed and calculated by the Beadview software. The lower and upper detection limits varied for specific proteins but were generally ~3.0 pg/ml and ~15,000 pg/ml respectively. Protein measurements above or below the limit of detection were imputed at that limit. Quality control of each sample was performed, and a bead count of <50 was not used for analysis.

### Data harmonization

The samples for each virus were obtained from three separate clinical studies, and CyTOF (i.e., surface marker quantification) was performed individually for each study. Thus, there was a risk of systematic bias due to batch effects in the raw surface marker data. To help minimize potential systematic bias, identification and quantification of each cell type was performed using manual gating on the combined data set. It is important to note that although the surface marker MFI values from CyTOF data are susceptible to batch effects, the cell-type abundance will be less affected since each cell type is clearly defined by expression of highly differentiating surface markers and assessed by an expert immunologist. To aid in detection of any serious batch effects, we included 4 CHIKV recovery samples as quality control (QC) that were repeatedly analyzed in all three virus batches during CyTOF data collection. Although we did observe some variance in cell type abundance estimates in the QC samples across batch, principal variance component analysis demonstrated that this variance was small relative to experimental factors such as time-point and virus. Thus, we decided against applying batch adjustment to the cell-type abundance data. We were not concerned about batch effects in the Luminex data since each experiment included a calibration curve using samples of known cytokine concentration to estimate protein concentration from MFI measurements, thus standardizing the data across experiments.

### Statistical analysis

All statistical analyses were performed using R (R-project.org) version 4.0.2 and available packages. Luminex data were analyzed as log_2_ of protein concentration obtained from the standard curve. CyTOF data were analyzed as log_10_ of cell-type frequency. CyTOF and Luminex data were modeled using linear mixed-effect models with gender, age, infection-stage (acute, recovery), and virus as fixed effects, including an infection-stage:virus interaction term and a random intercept across patientID. Models were fitted using the *limma* package/framework for high-throughput analysis. Hypotheses of interest were tested from the fitted model using contrasts and adjustment for multiple hypotheses across immune cell-types were carried out using the Benjamini-Hochberg procedure, which controls the FDR.

### DIABLO Analysis and visualization

DIABLO analysis was performed using the framework described in Singh et al, 2019 (24) using functions from the *mixOmics* R package [http://mixomics.org/]. In short, DIABLO extends sparse generalized canonical correlation analysis (sGCCA) into a supervised framework, identifying correlated components within and between each omic data type and the phenotype of interest. We performed DIABLO using inflammatory marker profiles (Luminex) and immune-cell profiles (CyTOF) for each patient, with virus (CHIKV, DENV, ZIKV) as the outcome variable. The first analysis used only the acute-phase measurements for each feature, whilst the second analysis used the difference between recovery and acute (recovery - acute). Network diagrams showing associations between component features were created using the ‘network’ function from the ‘mixOmics’ package and then exported to Cytoscape for further formatting. The ‘network’ function uses the methods described in González et al., 2012 (94).

## Data Availability

The datasets presented in this study can be found in online repositories. The names of the repository/repositories and accession number(s) can be found below: https://www.immport.org/home, SDY1288 and SDY1476.

## Supplementary material

Supplementary Figure 1 Quantification of immune-cell cytokine/chemokine/growth factor concentrations during acute arbovirus infection. Boxplots **(A-G)** show distribution of cytokine/chemokine/growth factor protein concentrations during acute infection with CHIKV, DENV, and ZIKV. Points indicate the mean protein concentration +95% CI, and asterisks above points indicate significant (FDR<0.05) concentration difference between viruses. *p<0.05; **p<0.01; ***p<0.001; ****p<0.0001.

Supplementary Figure 2 Frequency of NK cells expressing CD57 during acute infection with CHIKV, DENV and ZIKV and recovery phase. **(A)** Box plots show the percentage of NK cells expressing CD57 within the NK cell compartment during acute (left) and recovery (right) phase of infection with CHIKV, DENV and ZIKV. Colored circles indicate the mean frequency; asterisks indicate significant (FDR<0.05) differences between viruses. **(B)** Scatter plot shows changes in mean frequency (+/- 95% CI) of NK cells expressing CD57 during acute infection and recovery for each virus. Asterisks indicate significant difference between each pair of viruses *p<0.05; **p<0.01; ***p<0.001; ****p<0.0001.
