# Supplementary material for: Comparing human pediatric immune responses to primary infection with dengue, chikungunya and Zika viruses
Source: Front Immunol. 2025 Nov 19;16:1679566. doi: 10.3389/fimmu.2025.1679566 (PMC12672498; doi:10.3389/fimmu.2025.1679566)

Supplementary Material : Gating strategy for CyTOF data

Cell type definition

Cell Type	Definition — Gating strategy
Plasmacytoid dendritic cells	LIVE, CD3$^-$ CD19$^-$ CD56$^-$ CD16$^-$, CD66b$^-$ CD14$^-$, HLADR$^+$, CD123$^+$ CD11c$^-$
Myeloid dendritic cells (mDCs)	LIVE, CD3$^-$ CD19$^-$ CD56$^-$ CD16$^-$, CD66b$^-$ CD14$^-$, HLADR$^+$ CD11c$^+$
cDC1	mDCs, CD141$^+$ CD1c$^-$
cDC2	mDCs, CD141$^-$, CD1c$^+$
Classical monocytes	LIVE, CD3$^-$ CD19$^-$ CD56$^-$ CD16$^-$, CD66b$^-$, CD14$^+$ CD16$^-$
Intermediate monocytes	LIVE, CD3$^-$ CD19$^-$ CD56$^-$ CD16$^-$, CD66b$^-$, CD14$^+$ CD16$^+$
Non-classical monocytes	LIVE, CD3$^-$ CD19$^-$ CD56$^-$ CD16$^-$, CD66b$^-$, CD14low CD16$^+$
NK cells	LIVE, CD3$^-$ CD19$^-$ CD14$^-$, CD56$^+$ CD16$^{+/-}$
Basophils	LIVE, CD3$^-$ CD19$^-$ CD56$^-$ CD16$^-$, CD66b$^-$ CD14$^-$, HLADR$^-$ CD123$^+$
Neutrophils	LIVE, CD3$^-$ CD19$^-$, CD66b$^+$ CD14$^-$, HLADR$^-$ CD123$^-$, CD14$^-$ CD56$^-$
B cells	LIVE, CD3$^-$ CD19$^+$
Memory B cells	B cells, CD27$^+$ CD38$^-$
Naive B cells	B cells, CD27$^-$ CD38$^-$
Plasmablast and plasma B cells	B cells, CD27$^+$ CD38$^+$
T cells	LIVE, CD3$^+$ CD19$^-$
Activated T cells	T cells CD38$^+$, HLADR$^+$
CD4$^+$ T cells	T cells, CD4$^+$ CD8$^-$
Naive CD4 T cells	CD4 T cells, CD45RA$^+$ CD27$^+$
Central Memory CD4 T cells	CD4 T cells, CD45RA$^-$ CD27$^+$
Effector Memory CD4 T cells	CD4 T cells, CD45RA$^-$ CD27$^-$
TEMRA CD4 T cells	CD4 T cells, CD45RA$^+$ CD27$^-$
T follicular helper cells	CD4 T cells, CXCR5$^+$ CD27$^+$
CD8$^+$ T cells	T Cells, CD8$^+$ CD4$^-$
Naive CD8 T cells	CD8 T cells, CD45RA$^+$ CD27$^+$
Central Memory CD8 T cells	CD8 T cells, CD45RA$^-$ CD27$^+$
Effector Memory CD8 T cells	CD8 T cells, CD45RA$^-$ CD27$^-$
TEMRA CD8 T cells	CD8 T cells, CD45RA$^+$ CD27$^-$

Basophils

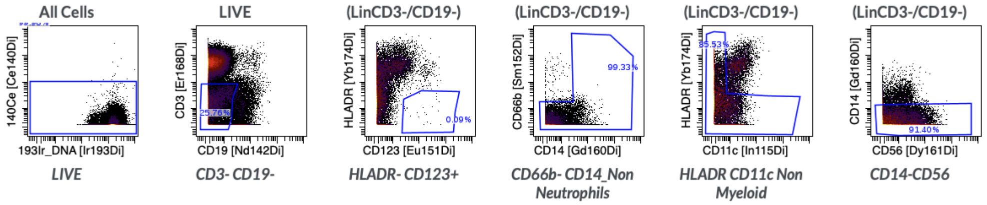

Neutrophils

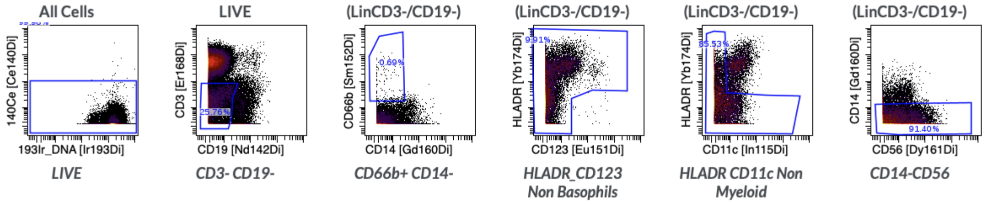

Dendritic cells

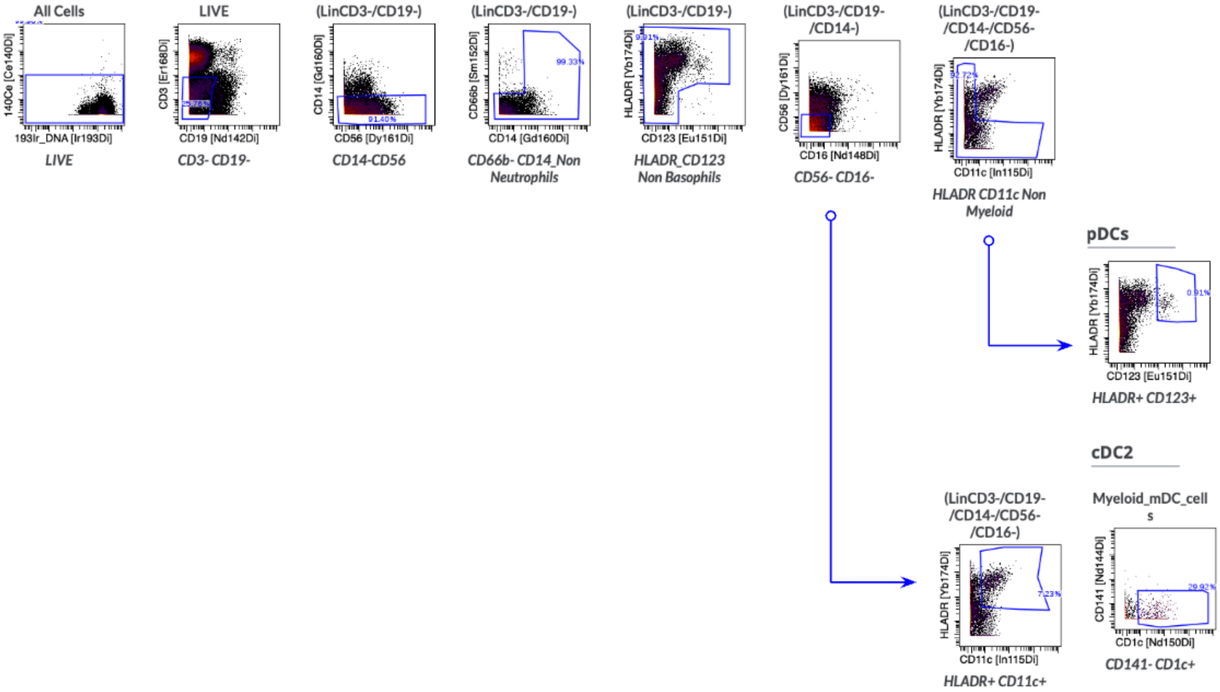

Monocytes

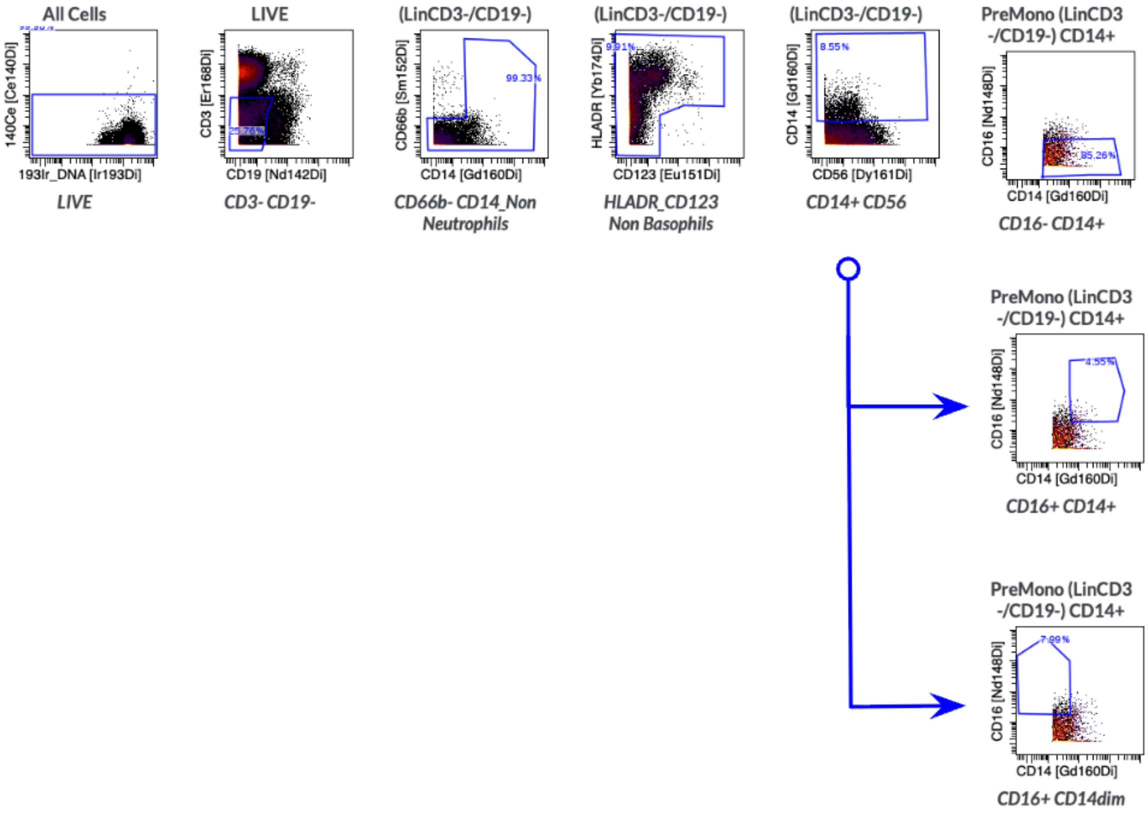
All Cells
140Ce [Ce140Di]
193Ir_DNA [Ir193Di]
LIVE
LIVE
CD3 [Er168Di]
CD19 [Nd142Di]
CD3- CD19-
(LinCD3-/CD19-)
CD66b [Sm152Di]
CD14 [Gd160Di]
99.33 %
CD66b- CD14_Non Neutrophils
(LinCD3-/CD19-)
HLADR [Yb174Di]
CD123 [Eu151Di]
HLADR_CD123 Non Basophils
(LinCD3-/CD19-)
CD14 [Gd160Di]
CD56 [Dy161Di]
8.55 %
CD14+ CD56
PreMono (LinCD3 -/CD19-) CD14+
CD16 [Nd148Di]
CD14 [Gd160Di]
85.26 %
CD16- CD14+
PreMono (LinCD3 -/CD19-) CD14+
CD16 [Nd148Di]
CD14 [Gd160Di]
4.55 %
CD16+ CD14+
PreMono (LinCD3 -/CD19-) CD14+
CD16 [Nd148Di]
CD14 [Gd160Di]
CD16+ CD14dim

NK cells

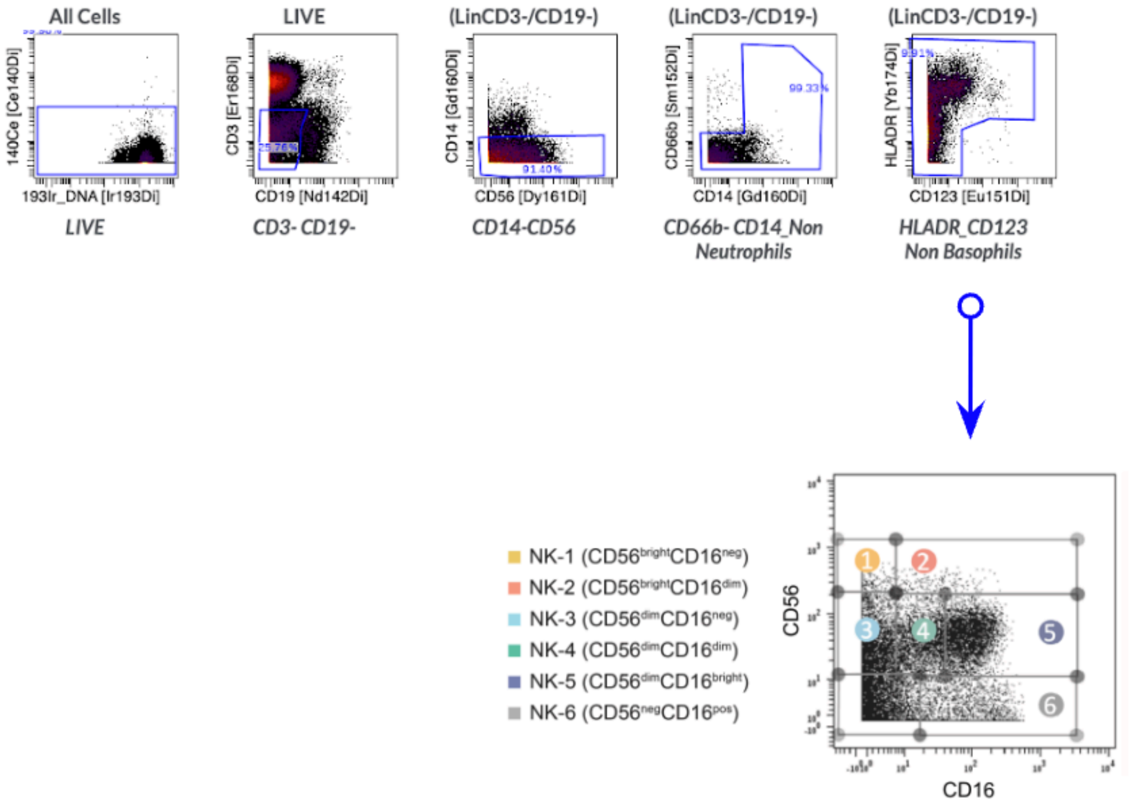

B cells

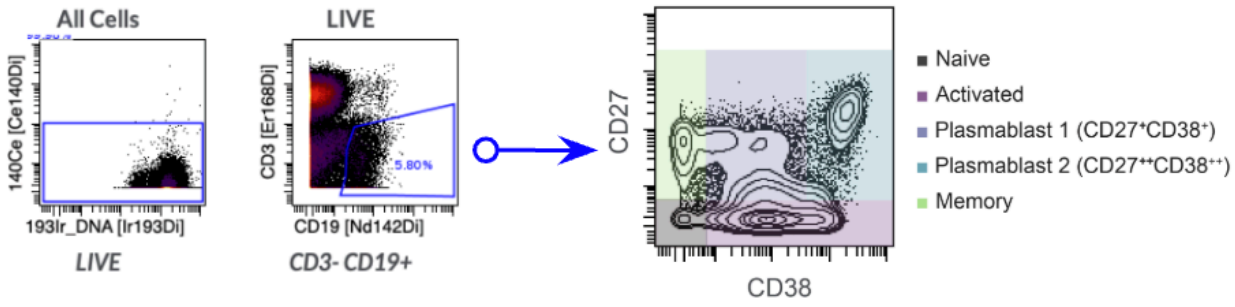

CD4 T Cells

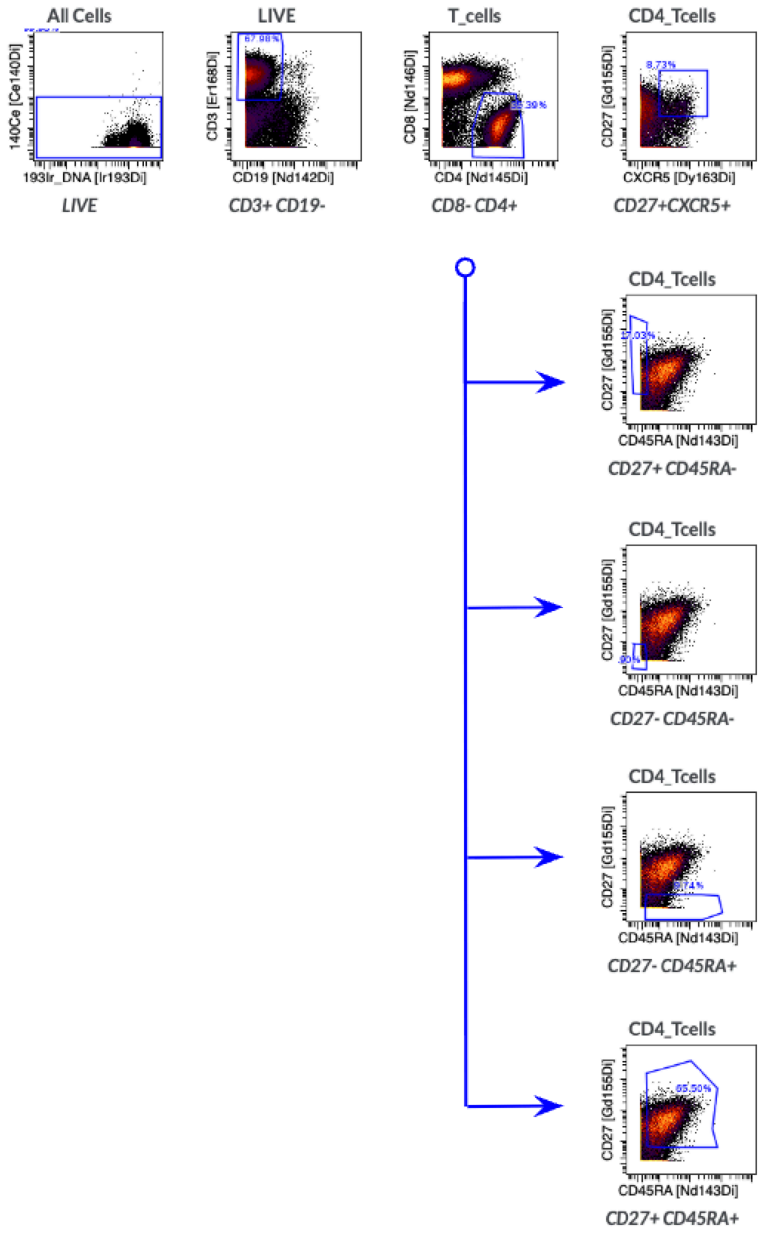

Activated CD4 T cells

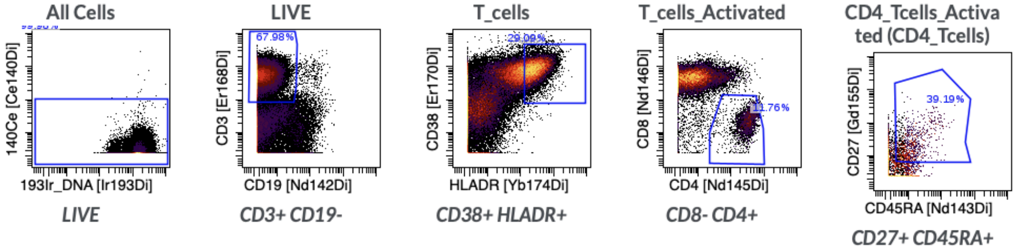

CD8 T Cells

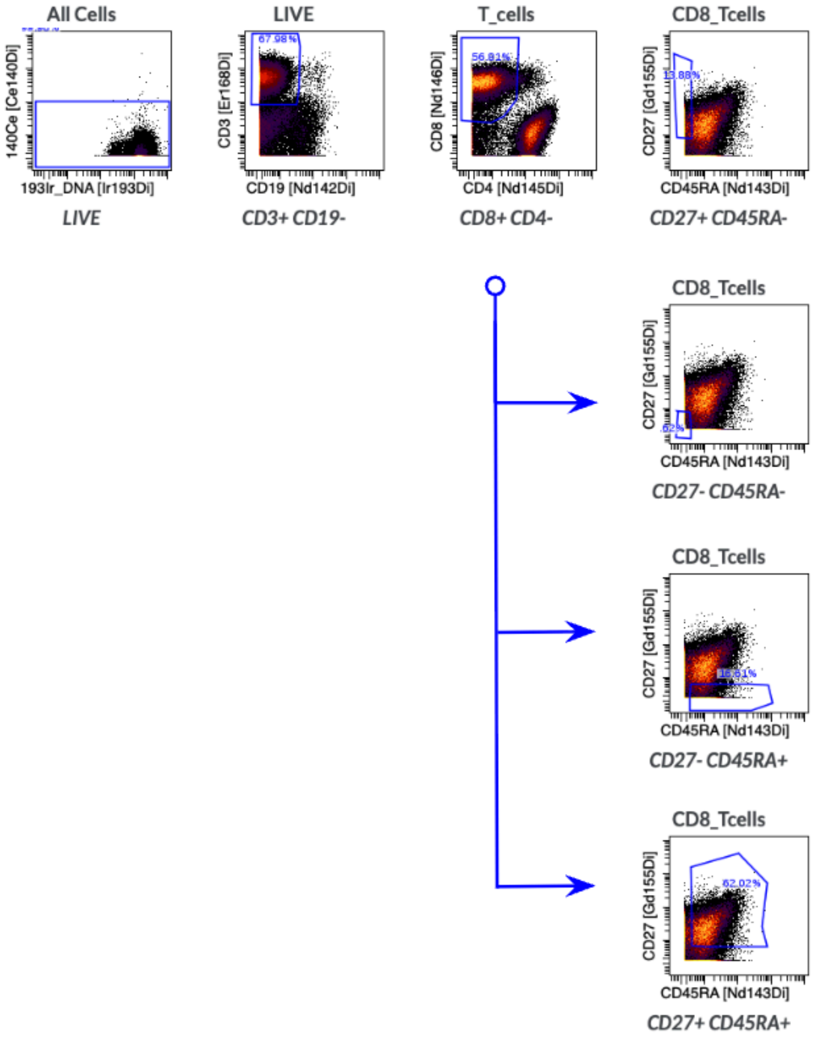

Activated CD8 T Cells

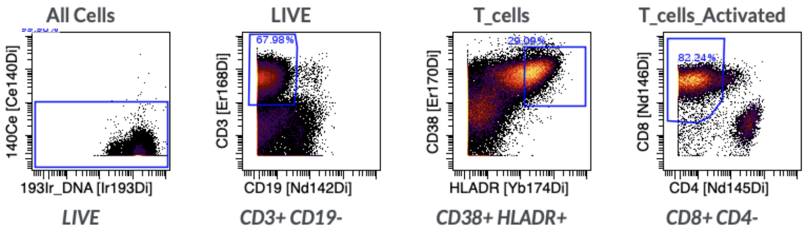

Supplement: Supplementary file 1 [file DataSheet1.pdf]
