# Supplementary figures and images for: Comparing human pediatric immune responses to primary infection with dengue, chikungunya and Zika viruses
Source: Front Immunol. 2025 Nov 19;16:1679566. doi: 10.3389/fimmu.2025.1679566 (PMC12672498; doi:10.3389/fimmu.2025.1679566)

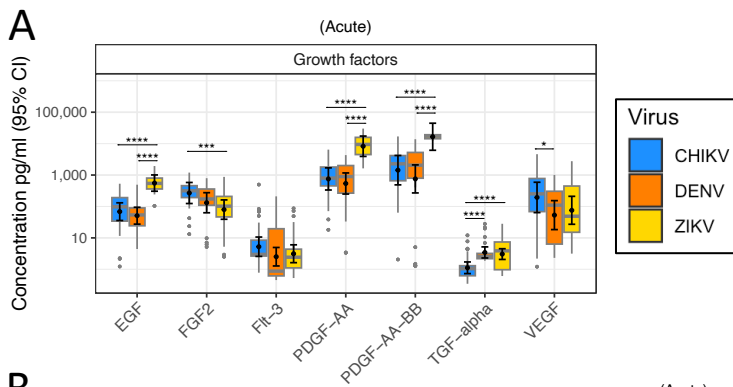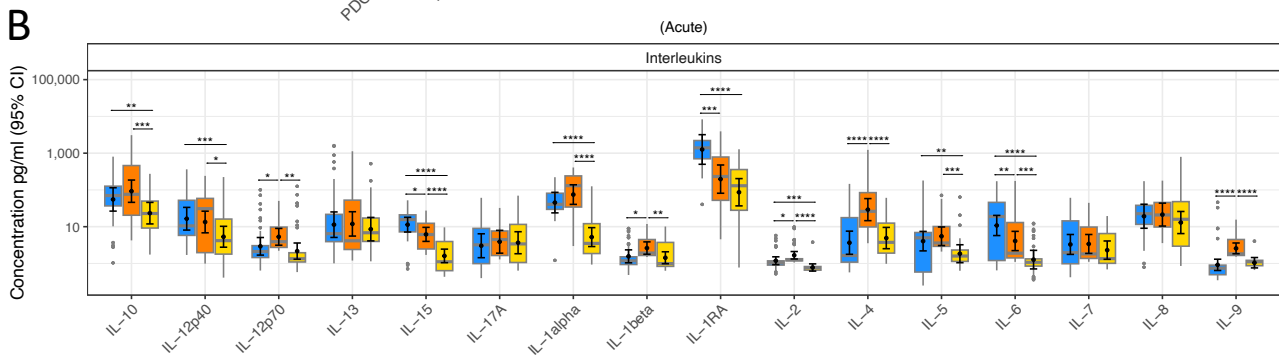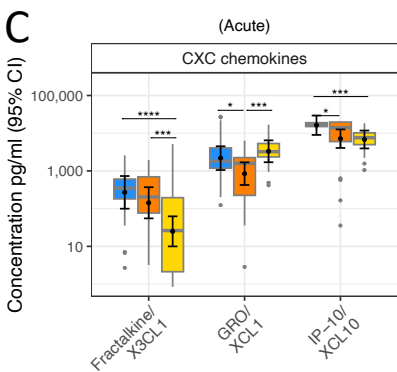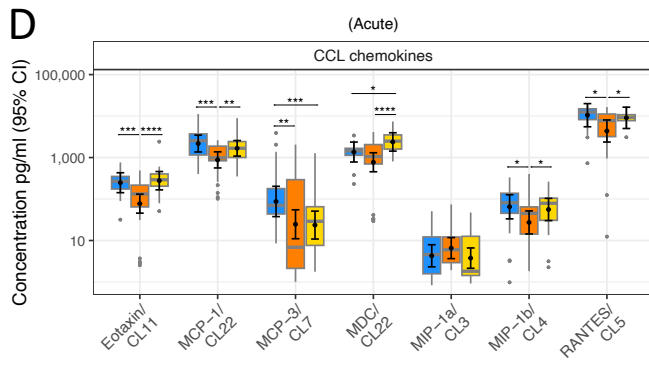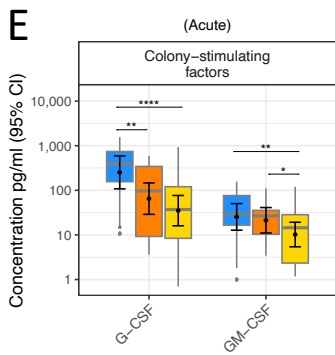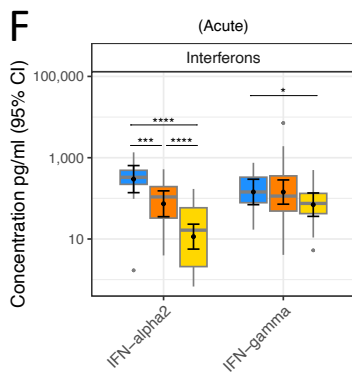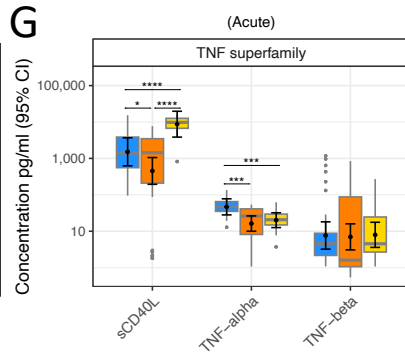

Supplement: Supplementary Figure 1 — Quantification of immune-cell cytokine/chemokine/growth factor concentrations during acute arbovirus infection. Boxplots (A-G) show distribution of cytokine/chemokine/growth factor protein concentrations during acute infection with CHIKV, DENV, and ZIKV. Points indicate the mean protein concentration +95% CI, and asterisks above points indicate significant (FDR<0.05) concentration difference between viruses. *p<0.05; **p<0.01; ***p<0.001; ****p<0.0001. [file Image1.pdf]

# NK cells CD57<sup>pos</sup>

**A**

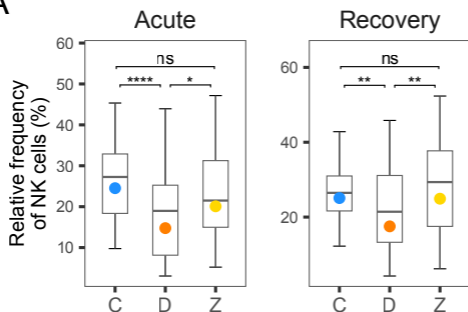

**B**

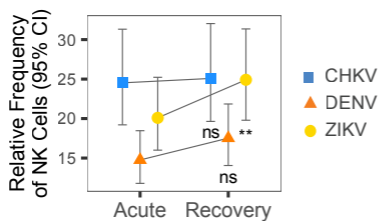

Supplement: Supplementary Figure 2 — Frequency of NK cells expressing CD57 during acute infection with CHIKV, DENV and ZIKV and recovery phase. (A) Box plots show the percentage of NK cells expressing CD57 within the NK cell compartment during acute (left) and recovery (right) phase of infection with CHIKV, DENV and ZIKV. Colored circles indicate the mean frequency; asterisks indicate significant (FDR<0.05) differences between viruses. (B) Scatter plot shows changes in mean frequency (+/- 95% CI) of NK cells expressing CD57 during acute infection and recovery for each virus. Asterisks indicate significant difference between each pair of viruses *p<0.05; **p<0.01; ***p<0.001; ****p<0.0001. [file Image2.pdf]
